# Geometric Microstructural Characteristics of White Matter Differentiate Patients With Facial Dyskinesias and Palsy

**DOI:** 10.1002/cns.70733

**Published:** 2026-01-28

**Authors:** Hua Zhu, Aocai Yang, Jixin Luan, Manxi Xu, Bing Liu, Kuan Lv, Pianpian Hu, Amir Shmuel, Haogang Zhu, Zhen Yuan, Ni Shu, Jian Cheng, Guolin Ma

**Affiliations:** ^1^ Beijing Advanced Innovation Center for Biomedical Engineering, School of Biological Science and Medical Engineering Beihang University Beijing China; ^2^ State Key Laboratory of Complex & Critical Software Environment, School of Computer Science and Engineering Beihang University Beijing China; ^3^ International Innovation Institute Beihang University Hangzhou China; ^4^ Department of Radiology China‐Japan Friendship Hospital Beijing China; ^5^ China‐Japan Friendship Hospital (Institute of Clinical Medical Sciences) Chinese Academy of Medical Sciences & Peking Union Medical College Beijing China; ^6^ Peking University China‐Japan Friendship School of Clinical Medicine Beijing China; ^7^ McConnell Brain Imaging Centre, Montreal Neurological Institute McGill University Montreal Quebec Canada; ^8^ Departments of Neurology and Neurosurgery, Physiology, and Biomedical Engineering McGill University Montreal Quebec Canada; ^9^ Center for Cognitive and Brain Sciences University of Macau Macau SAR China; ^10^ State Key Laboratory of Cognitive Neuroscience and Learning & IDG/McGovern Institute for Brain Research Beijing Normal University Beijing China; ^11^ BABRI Centre Beijing Normal University Beijing China; ^12^ Beijing Key Laboratory of Brain Imaging and Connectomics Beijing Normal University Beijing China

**Keywords:** diffusion tensor imaging, facial palsy, facial paralysis, Meige's syndrome

## Abstract

**Purpose:**

The heterogeneous and homogeneous clinical manifestations of peripheral facial palsy (FP), hemifacial spasm (HFS), and Meige's syndrome (MS) complicate the differentiation of diagnoses for these facial motor diseases. To comprehensively investigate the white matter microstructural characteristics in patients with facial dyskinesias and palsy using geometric and integrity metrics in DTI.

**Material and Methods:**

In this prospective study conducted from September 2020 to January 2022, patients with FP, HFS, and MS, as well as sex‐matched healthy control subjects, underwent 3.0 T MRI. Geometric metrics (i.e., splay, bend, twist, and total distortion) based on “Director Field Analysis” and fractional anisotropy (FA) and mean diffusivity (MD) were calculated from DTI data. Cross‐sectional tract‐based spatial statistics were performed among FP, HFS, MS patients, and healthy controls. The correlation between disease severity and DTI metrics was evaluated. Additionally, the geometric microstructural properties combining FA and MD were used to classify FP, HFS, and MS patients using machine learning methods.

**Results:**

Geometric metrics and FA/MD were widely altered across white matter in FP and HFS patients compared with healthy controls. However, in MS patients only DFA metrics were significantly altered. FA and DFA values strongly correlated with the severity of facial movement disorder in FP patients. Combing conventional FA/MD value with DFA metrics enabled the diagnostic differentiation of FP and HFS from MS.

**Conclusion:**

Our findings demonstrated that the geometric microstructural information of white matter fibers could provide novel insight into the underlying pathological changes in facial dyskinesias and palsy.

## Graphical abstract

Patients with facial dyskinesias and palsy show distinct white matter geometric microstructural patterns. Meige's syndrome patients exhibit markedly altered white matter integrity and geometry, while facial palsy patients' white matter features correlate with clinical severity. Integrating traditional Diffusion Tensor Imaging measurements (fractional anisotropy and mean diffusivity) and Director Field Analysis‐based geometric metrics improves discrimination between facial dyskinesias and palsy.

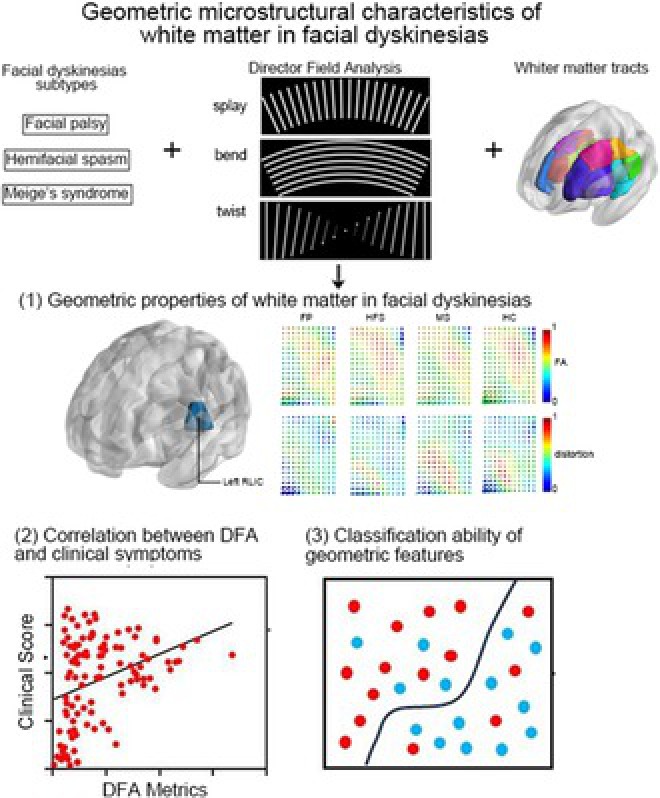

## 1 Introduction

Facial dyskinesias and palsy encompass a spectrum of motor disorders that affect the face, typically characterized by focal muscle weakness and dystonia. Peripheral facial palsy (FP) is a common condition marked by facial muscle weakness or paralysis resulting from damage to the facial nerve [1]. Among the complications of facial palsy, facial dyskinesias are particularly significant. As the primary motor nerve responsible for innervating the facial muscles, the facial nerve plays a crucial role in regulating facial symmetry, expression, and movement [2]. Approximately 80% of peripheral FP cases are classified as idiopathic, meaning the underlying cause is unknown. Hemifacial spasm (HFS) is another neuromuscular disorder characterized by involuntary clonus or tonic contractions of muscles in the upper or lower face, including the platysma muscle [3]. HFS typically initiates around the eyes, manifesting symptoms such as involuntary eyelid closure and raised eyebrows, before spreading to involve the entire face. HFS presents obvious facial dystonia in facial dyskinesias. The primary cause of HFS is often attributed to vascular compression at the exit zone of the seventh cranial nerve in the pontine exit area. Meige's syndrome (MS) is a form of cranial dystonia characterized by blepharospasm (involuntary eyelid closure) and oromandibular dystonia, with its pathological mechanism still unclear [4]. The diverse clinical presentations of FP, HFS, and MS, along with the overlap between HFS and MS in terms of facial dystonia disorders, pose challenges in their clinical differentiation.

Moreover, the neurological mechanisms underlying these conditions remain ambiguous. In recent years, there has been a shift toward the neural plasticity interpretation of neurocircuits and networks in the common understanding of the pathophysiology of these diseases [5, 6, 7]. While FP and HFS are classically considered disorders of the peripheral nervous system, emerging evidence from structural and functional MRI studies indicates concomitant and potentially compensatory/reorganizational changes within the central nervous system [8, 9, 10] (cite references). Similarly, the pathophysiology of MS is thought to involve dysfunctional circuits within the basal ganglia and thalamocortical networks. Therefore, investigating white matter microstructure—which forms the structural backbone of these large‐scale neural networks—provides a crucial window into understanding the central mechanisms that may underlie, modulate, or result from these facial movement disorders, potentially offering new insights beyond peripheral pathology.

Advanced neuroimaging techniques have provided insights into the structural and functional abnormalities in the central nervous system associated with these conditions. Diffusion Tensor Imaging (DTI) is a commonly used method to identify white matter microstructural changes in the brain by measuring the diffusion properties of water molecules. Various metrics can be derived to describe the integrity and organization of white matter fiber bundles. Numerous DTI‐based HFS studies have reported reductions in white matter microstructural integrity as indicated by voxel‐based metrics [11]. Previous studies demonstrated that brain structure abnormality in facial muscles dystonia and palsy can, in turn, further entrench the disorder [12]. However, the common limitations of previous DTI‐based studies are unable to model voxels containing multiple fiber populations, and classical metrics cannot display the directional diffusion from the intra‐voxel analysis. Nowadays, a novel DTI metric called Director Field Analysis (DFA) has been proposed to study the orientational distortion (i.e., splay, bend, twist, and total distortion) of local white matter. From the view of orientational properties hidden in neighboring inter‐voxel information, DFA metrics provide additional information about microstructural changes in the white matter and can help understand the underlying mechanisms of these disorders [13]. In contrast to conventional intravoxel‐based metrics like fractional anisotropy (FA) and mean diffusivity (MD), which provide information within a voxel, DFA enables the measurement of orientational geometric changes in local white matter fibers across voxels. However, few studies are using DFA to characterize brain geometric microstructural characteristics in patients with facial paralysis, hemifacial spasm, and Meige's syndrome. And we firmly believe that unraveling the integrated microstructure changes in white matter holds the key to understanding the CNS mechanism of those facial disorders.

Therefore, in this study, we present the first application of geometric microstructural information to assess the orientation of local white matter fibers in individuals with FP, HFS, and MS. Our objective was to evaluate the specific pattern of orientational and integrity changes in local white matter fibers among these conditions and to explore the diagnostic potential of DFA in their differentiation.

## 2 Materials and Methods

This study was approved by the Ethics Committee of the China‐Japan Friendship Hospital (Approval ID: 2020‐KY‐181). All the participants and/or their relatives were informed about this study and provided their written informed consent.

### 2.1 Participants

From September 2020 to January 2022, patients diagnosed with FP, HFS, and MS were recruited from the Department of Neurosurgery at China‐Japan Friendship Hospital. First‐ever idiopathic unilateral FP and primary HFS were diagnosed by two experienced neurologists. Primary MS patients were diagnosed based on combined clinical features of facial dystonia and history [4]. The inclusion criteria of FP, HFS, and MS patients were as follows: (I) age > 18; (II) no treatment within the past 3 months; (III) right‐handed; and (IV) complete MRI data. The Toronto Facial Grading System (TFGS) [14] score was employed to rate facial nerve dysfunction in FP patients. The TFGS test comprises three sub‐scores for each division of the face, including resting symmetry score (TFGS_1), symmetry of voluntary movement score (TFGS_2), and synkinesis score (TFGS_3). The Cohen spasm scale [15] was used to rank the spasm severity of HFS patients. The Burke‐Fahn‐Marsden dystonia rating scale‐movement (BFMDRS‐M) [16, 17] was employed to evaluate the dystonia impairment in MS patients, with the BFMDRS_eye sub‐score used to assess the severity of dystonia in the eyes. The blepharospasm disability index (BSDI) scale was used to measure impairment of daily activities caused by blepharospasm [18]. The rater of the above clinical tests was blinded to the disease status and neuroimaging results. Additionally, healthy controls (HC) were recruited from the local communities simultaneously. As the mean age of the MS group was older than FP and HFS, HCs were randomly divided into HC_1 and HC_2 groups to match age and sex in different patient groups. The HC_1 was used for comparison with FP and HFS patients, and HC_2 was used for comparison with MS patients. The exclusion criteria for all participants were (I) other neurologic and psychiatric diseases; (II) metabolic disease; (III) alcohol or drug abuse; (IV) MRI contraindications; and (V) abnormal findings on brain imaging.

### 2.2 MRI Data Acquisition

MR examinations were conducted on a 3.0 T MR scanner (Discovery MR750 scanner; GE Medical Systems, USA) with eight‐channel head coils. The scanning protocol included spin‐echo echo‐planar sequence for the diffusion images, and the acquisition parameters were as follows: repetition time = 8028 ms, echo time = 81.8 ms, flip angle = 90°, slice thickness = 2 mm, field of view = 240 × 240 mm^2^, voxel size = 2 × 2 × 2 mm^3^, 64 encoding directions (b = 1000 s/mm^2^), and 8 non‐diffusion‐weighted images (b = 0 s/mm^2^).

Conventional MR sequences (3D‐T1, T2WI, T2‐FLAIR) were also included to detect brain abnormalities. All raw image data were visually examined to exclude subjects with visible image artifacts.

### 2.3 Data Processing

**FIGURE 1:**
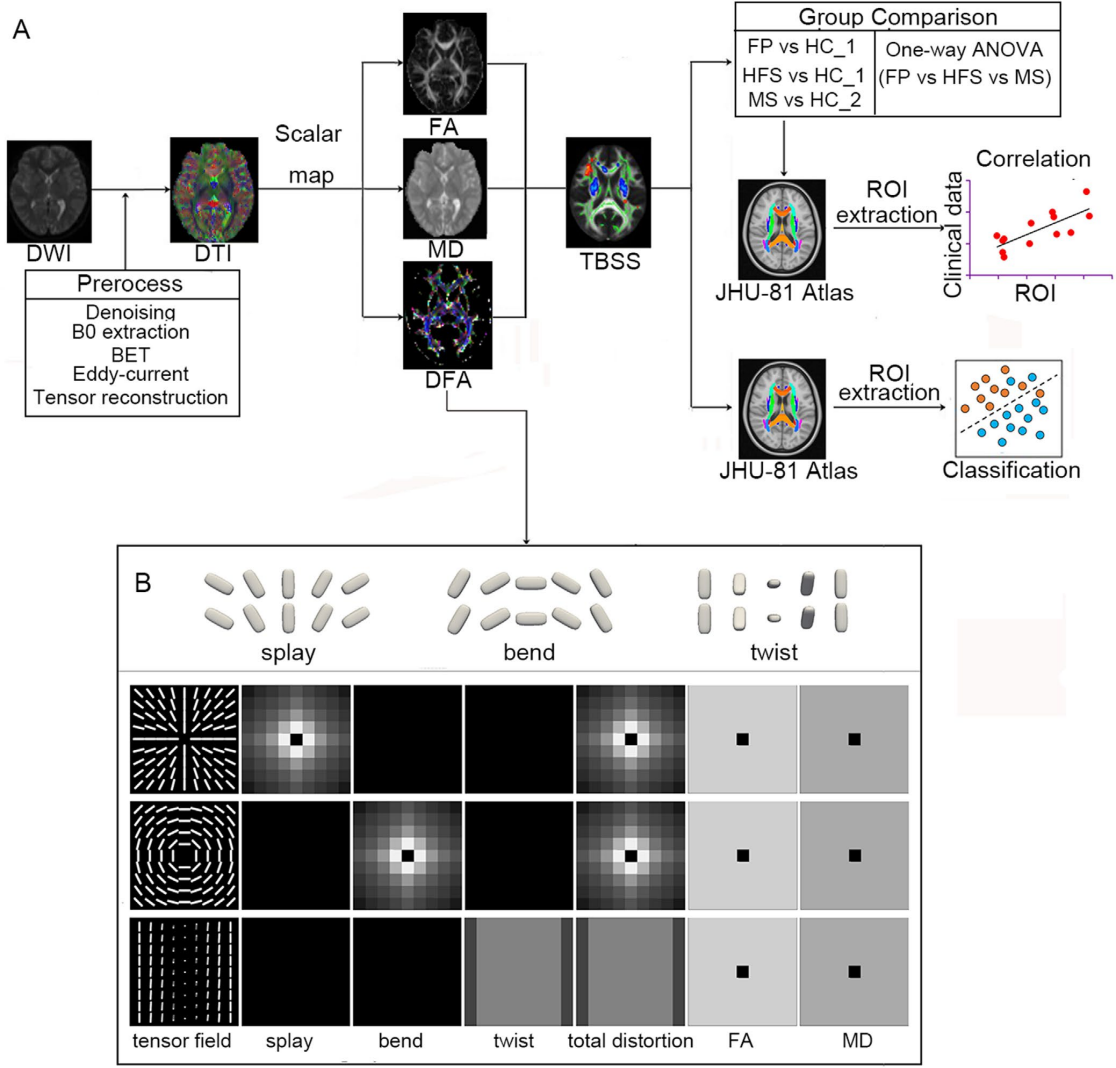
Methodological illustration. (A) Schematic representation of a general pipeline. Briefly, diffusion weighted images (DWI) were preprocessed and diffusion tensor models (DTI) were reconstructed. Then scalar maps (e.g., FA, MD, DFA indices) were calculated and tract‐based spatial statistics (TBSS) was performed for statistical analysis. (B) A brief illustration of three types of orientational distortions (i.e., splay, bend, and twist) in part 1. The figure was revised and approved from Cheng and Basser (2017) [13]. In part 2, each row shows a synthetic tensor field with DFA index maps (splay, bend, twist, and total distortion) calculated from the tensor field. The first row shows tensor orientations splay around the central voxel in a tensor field. The splay index quantified the degree of splay. The second row shows tensor orientations bend around the central voxel in a tensor field. The bend index quantifies the degree of bend. The third row shows a twist pattern, where from left to right in each row a tensor twists its orientation around the left–right axis. The twist index map is constant for this tensor field, ignoring the boundary effect in the calculation of the boundary voxels of the field. Although in these three tensor fields, only one index map among splay, bend and twist maps is nonzero, these three index maps are normally all nonzero for real data, because the spatial orientational distortion normally happens in three‐dimensional space for real data, not in a two‐dimensional space.

The analysis pipeline was shown in Figure 1A.

#### 2.3.1 Preprocessing of Diffusion Data

To standardize the affected side across patients, diffusion images from left‐affected HFS and FP patients were flipped from left to right. In this study, the “right” side was defined as the affected side for both FP and HFS, while the “left” was considered the contralateral side. The images of healthy controls were not flipped. The diffusion data were then denoised using MRtrix3 toolbox [19]. The Brain Extraction Tool (BET) [20] in FSL (fMRIB Software Library, version 6.0.4, www.fmrib.ox.ac.uk) was used to remove non‐brain tissue from the mean B0 image followed by eddy current correction [21] on the remaining diffusion data. The DMRITool (http://diffusionmritool.github.io) was utilized to reconstruct diffusion tensors and calculate DTI metric maps for each subject, including FA, MD, and DFA metrics (splay, bend, twist, and total distortion) [13].

#### 2.3.2 Calculation of DFA Metrics

A novel mathematical framework of DFA was employed to compute the orientational distortion of local white matter regions based on tensor images. Based on studies of liquid crystals, three types of orientational distortions are proposed:

1. Splay: spatial bending occurs perpendicular to the direction of the main molecular axis.
2. Bend: spatial bending is parallel to the direction of the main molecular axis
3. Twist: neighboring directions are rotated with respect to one another, rather than aligned.

Figure 1B shows three synthetic tensor fields with their six index maps (splay, bend, twist, total distortion, FA and MD). The fundamental concept of DFA involves constructing a local orthogonal frame at each voxel, with the first axis representing the main direction of that voxel. Subsequently, inter‐voxel DFA metrics (splay, bend, twist) are defined using spatial derivatives of the main direction along different axes. Drawing an analogy from liquid crystal physics, in the context of white matter, “splay” may reflect fanning or divergence of fiber bundles; “bend” may indicate sharp curvature or winding of a fiber tract; and “twist” might be associated with the lateral torsion or crossing of neighboring fibers. The total distortion metric is computed as the following formula: $\text{total distortion}=\sqrt{{\text{splay}}^{2}+{\text{bend}}^{2}+{\text{twist}}^{2}}$. DFA metrics serve to quantify the local geometric microstructures in the orientation of white matter within a local neighborhood at an inter‐voxel level. See Figure 1. The first row in Figure 1B shows a tensor field with a circular splay pattern, where tensor orientations splay around the central voxel. Ignoring the singularity of the central voxel, both the bend and twist indices are zero, and the splay index quantifies the degree of splay. The second row in Figure 1B shows a tensor field with a circular voxel. Both the splay and twist indices are zero, and the bend index quantifies the degree of bend. The third row in Figure 1B shows a twist pattern, where from left to right in each row a tensor twists its orientation around the left–right axis. The twist index map is constant for this tensor field, ignoring the boundary effect in the calculation of the boundary voxels of the field, while both splay and bend maps are zero. Although in these three tensor fields, only one index map among splay, bend, and twist map is nonzero, these three index maps are normally all nonzero for real data, because the spatial orientational distortion normally happens in the three‐dimensional space for real data, not in a two‐dimensional space. The DFA metrics, alongside the conventional FA or MD metrics that measure white matter integrity, expand and enhance the characterization of white matter microstructure by incorporating both integrity and geometrics. Detailed interpretations for DFA metrics have been described in previous study [13].

### 2.4 Statistical Analysis

#### 2.4.1 Tract‐Based Spatial Statistics (TBSS)

The FA map for each subject was nonlinearly registered to the FMRIB58_FA 1 mm template using FSL's FNIRT. Subsequently, the group mean FA image was generated following the standard TBSS pipeline: (i) eroding the mean FA image; (ii) thresholding at FA > 0.2; (iii) thinning to 1‐voxel width using medial axis transform; and (iv) masking with b0 image. Next, individual participant FA data were then projected onto this mean FA skeleton. Additionally, the transformation relationship derived from the non‐linear registration of FA images was utilized to warp DFA metrics and MD image. The warped DFA and MD maps were further projected onto the group‐specific white matter skeleton.

Voxel‐wise statistical analyses of FA, MD, and DFA metrics were conducted on the white matter skeleton in FSL. General linear models were generated for group comparisons, with age and sex considered as nuisance covariates. Randomized permutation nonparametric tests (10,000 permutations) with the threshold‐free cluster enhancement algorithm (TFCE) were employed to assess group differences in FA, MD, and DFA metrics between FP, HFS, MS, and HC. Furthermore, randomize statistics were performed to assess group differences among FP, HFS, and MS patients. The statistical significance threshold was set at a family‐wise error (FWE)‐corrected *p* < 0.05.

Additionally, to assess the age effect between two groups of healthy controls (HC_1 and HC_2), we conducted the TBSS analyses of FA, MD, and DFA metrics.

#### 2.4.2 Correlation Analysis Between White Matter Microstructure and Clinical Tests

Following the TBSS analysis of patients and HC, we extracted the mean values of FA, MD, and DFA from the white matter tracts exhibiting significant clusters (voxel number > 50) using FSL's fslmeants. These tracts were identified using the JHU‐81 atlas within FSL. The Spearman's correlation analyses were performed between clinical tests (disease duration, sub‐scores, and total scores of TFGS for FP, Cohen scores for HFS patients, BFMDRS‐M, and BDSI for MS patients) and the white matter microstructure metrics (FA, MD, and DFA) of white matter tracts exhibiting significant changes in TBSS. To correct for multiple comparisons, the false discovery rate (FDR) method with a significance threshold of *p* < 0.05 was applied.

#### 2.4.3 Classification Among FP, HFS, MS and HC

To validate the classification capabilities of FA, MD, and DFA metrics in facial dyskinesias and palsy, we introduced AutoGluon (version 0.5.2) for automated design and use of machine learning models for classification tasks. AutoGluon employs a model stacking approach to enhance predictive performance by combining the strengths of multiple base models. This method leverages a diverse set of algorithms, such as CatBoost, LightGBM, and neural networks, to generate robust predictions. The stacking technique reduces the risk of overfitting by balancing the biases and variances of different models, leading to improved generalization on unseen data. Moreover, AutoGluon's automation of the stacking process simplifies model selection and integration, making it accessible for users to achieve high accuracy with minimal manual intervention. In the current version of AutoGluon, the selected models include CatBoost model, Extra Trees model, KNearestNeightbors model, LightGBM model, NeuralNet model, Linear model, Random Forest model, and XGBoost model.

In this study, initially, we first selected all 48 white matter regions in JHU‐81 atlas as regions of interest (ROIs) and extracted the mean values of FA, MD, and DFA metrics within these white matter ROIs as features for each patient. Three distinct feature sets were developed for model training:

1. The first set consisted of FA/MD metrics arranged into an array of size N_subj_ by 96.

2. The second set comprised DFA metrics organized into an array of size N_subj_ by 192.

3. The third set was a comprehensive array that combined FA/MD and DFA metrics, with a size of N_subj_ by 288.

A 10‐fold cross‐validation framework was applied to evaluate the performance of each training model. For each training model, the whole corresponding feature vector was randomly split into 10 folds. In every fold, one of the 10 parts was regarded as the test set, and the rest as the training set. Machine learning models training and hyper‐parameter tuning were performed with AutoGluon's Tabular Prediction function on the training set. Then, deployment and application of the training model were performed with the test set, and the true positive rate and the false positive rate were obtained based on the true labels and corresponding predicted probabilities generated in each fold. Then we calculated the average true positive rates (TPR) and false positive rates (FPR) across the 10 folds. The receiver operating characteristic (ROC) curve was plotted using average TPR and FPR values, and the area under the curve (AUC) score was calculated for model verification and evaluation. Additionally, multi‐classification for three types of facial dyskinesias and palsy (FP, HFS, and MS) was performed, and the confusion matrix was used to evaluate the classification performance using all metrics features, FA/MD metrics features, and DFA metrics features, respectively. Also, we conducted analyses comparing AutoGluon with standalone SVM (RBF kernel), logistic regression (L2 regularization), and random forest (100 trees) implementations.

#### 2.4.4 Demographic and Clinical Data

Additional statistical analyses were conducted using SPSS version 21.0. Inter‐group comparisons of age and educational level were performed using independent t‐tests. Differences between groups stratified by sex were assessed using the Chi‐square (χ2) test. The *p* < 0.05 was considered statistically significant.

## 3 Results

### 3.1 Clinical and Demographic Characteristics

In this study, a total of 50 FP patients, 57 HFS patients, 31 MS patients, and 55 HC were finally included. No significant differences in sex or age were observed between the FP, HFS, MS groups, and their corresponding HCs (*p* > 0.05). However, the education level was found to be lower in the HFS group compared to the HC group (*p* = 0.001). The demographic and clinical information is summarized in Table 1.

**TABLE 1:** Clinical and Demographic Characteristics.

Characteristic	HC_1 (*n* = 33)	HC_2 (*n* = 30)	FP (*n* = 50)	HFS (*n* = 57)	MS (*n* = 31)	*P*_HC_1 VS FP_	*P*_HC_1 VS HFS_	*P*_HC_2 VS MS_
Sex (M/F)^†^	13/20	10/20	18/32	12/45	8/23	0.82	0.08	0.58
Age (y)^††^	46.6 ± 14.5	63.4 ± 7.7	40.9 ± 15.3	51.1 ± 10.4	58.3 ± 9.6	0.2	0.17	0.19
Education (y)^††^	14.1 ± 4.4	9.0 ± 4.1	12.2 ± 4.2	10.6 ± 3.9	8.2 ± 4.8	0.11	0.001***	0.48
Affected side (L/R)	/	/	24/26	30/27	/	/	/	/
TFGS	/	/	18.6 ± 17.2	/	/	/	/	/
TFGS_1	/	/	16.5 ± 3.7	/	/	/	/	/
TFGS_2	/	/	39.0 ± 18.2	/	/	/	/	/
TFGS_3	/	/	3.9 ± 5.9	/	/	/	/	/
Cohen	/	/	/	2.8 ± 0.8	/	/	/	/
BFMDRS_M	/	/	/	/	7.6 ± 2.6	/	/	/
BFMDRS_eye	/	/	/	/	4.9 ± 1.9	/	/	/
BSDI	/	/	/	/	12.3 ± 5.6	/	/	/

*Note:* Data were shown as mean ± standard deviation.

Abbreviations: BFMDRS_eye, blepharospasm score of BFMDRS; BFMDRS_M, Burke‐Fahn‐Marsden dystonia rating scale‐movement; BSDI, blepharospasm disability index; F, female; L, left side; M, male; R, right side; TFGS, Toronto Facial Grading System; TFGS_1, resting symmetry score of TFGS; TFGS_2, symmetry of voluntary movement score of TFGS; TFGS_3, synkinesis score of TFGS.

^†^ Chi‐square (χ^2^) test.

^††^ Independent t‐test.

*** *p* < 0.001.

### 3.2 Group Differences of FA/MD and DFA Between FP, HFS, MS and HCs

**FIGURE 2:**
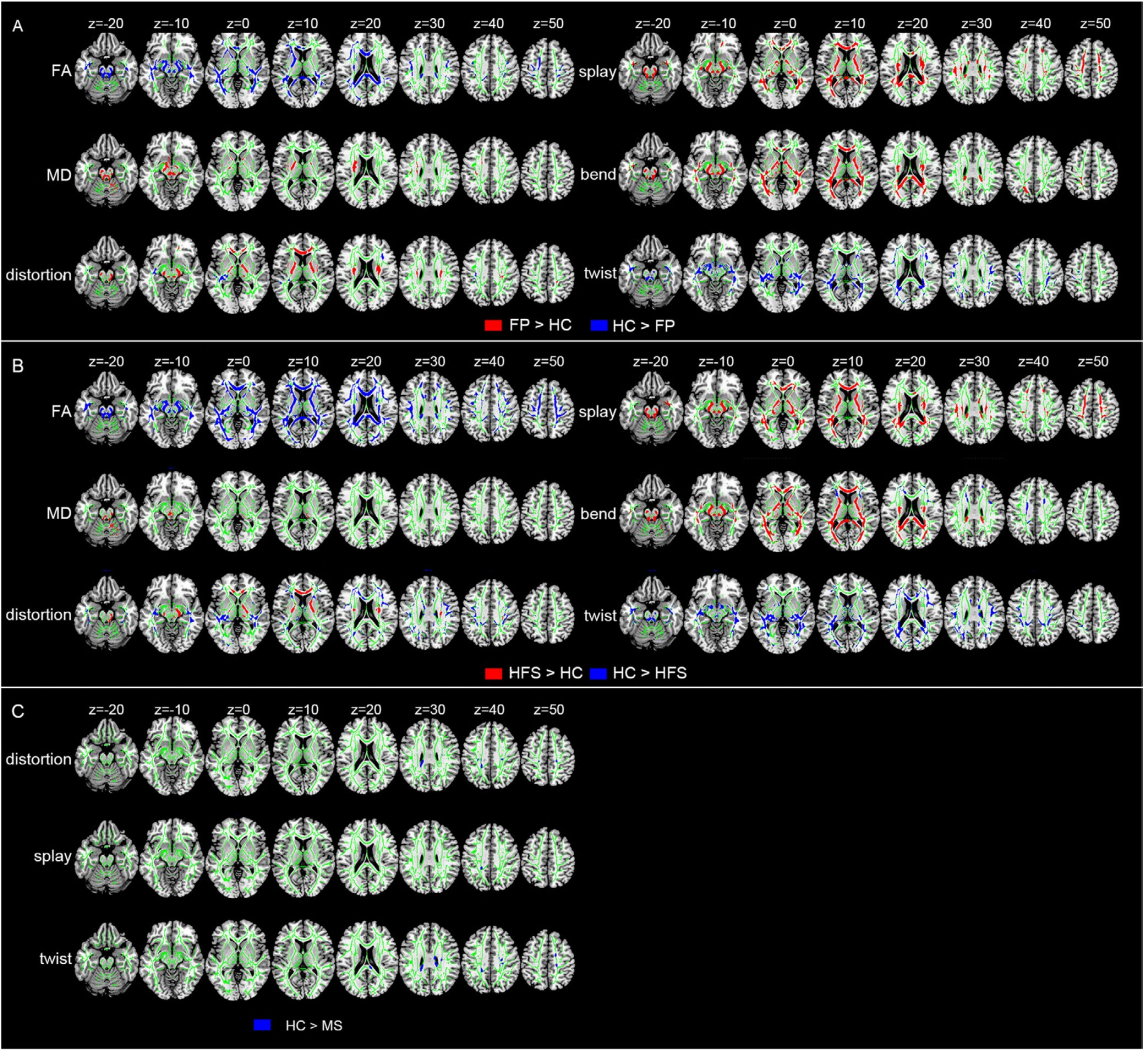
Microstructural changes in the orientation (i.e., splay, bend, twist, total distortion) and integrity (i.e., FA, MD) between FP patients and HC_1, HFS patients and HC_1, MS patients and HC_2 across the whole brain white matter. (A) Changes in the orientation and integrity in the FP patients compared to HC_1. (B) Changes in the orientation and integrity in the HFS patients compared to HC_1. (C) Changes in the orientation in the MS patients compared to HC_2. Significant voxels (*p* < 0.05, corrected for multiple comparisons) were thickened by using the TBSS fill function into local tracts (red/blue) and overlaid on the white matter skeleton (green). Red denotes the regions in facial dyskinesias patients larger than in healthy controls. Blue denotes the regions in facial dyskinesias patients less than in healthy controls. A complete listing of all significant white matter tracts is provided in Tables S2–S4.

Group differences in FA, MD, and DFA metrics across the whole brain between facial diseases and HCs were shown in Figure 2. The abbreviations of white matter ROIs were summarized in Table S1. We listed the top 5 tracts with the most voxels in each cluster.

Compared with HCs, distinct differences in the microstructure of white matter were observed in FP patients, characterized by a decrease in FA and total distortion, as well as an increase in MD, splay, bend, and total distortion in the corpus callosum, cerebellar peduncle, thalamic radiata, internal capsule, external capsule, and corona radiata. Specifically, FP patients exhibited specific elevation of twist in the fornix, sagittal striatum, and superior longitudinal fasciculus compared to HCs (Figure 2A and Table S2).

Additionally, HFS patients demonstrated significant discrepancies in white matter microstructure, including decreased FA, total distortion, and twist, as well as increased MD, total distortion, splay, and bend in the corpus callosum, cerebellar peduncle, corona radiata, internal capsule, and corona radiata compared to HCs. Notably, HFS patients also showed notable alterations in DFA metrics (increase in splay, bend, decrease in twist) in the fornix, thalamic radiata, superior longitudinal fasciculus, sagittal stratum, and external capsule, along with a reduction in MD in the pontine crossing tract and corticospinal tract (Figure 2B and Table S3).

Lastly, MS patients exhibited a significant decrease in DFA metrics (total distortion, splay, and twist) in the corpus callosum and corona radiate compared to HCs (Figure 2C) and Table S4.

### 3.3 Group Differences of FA/MD and DFA Among FP, HFS and MS

**FIGURE 3:**
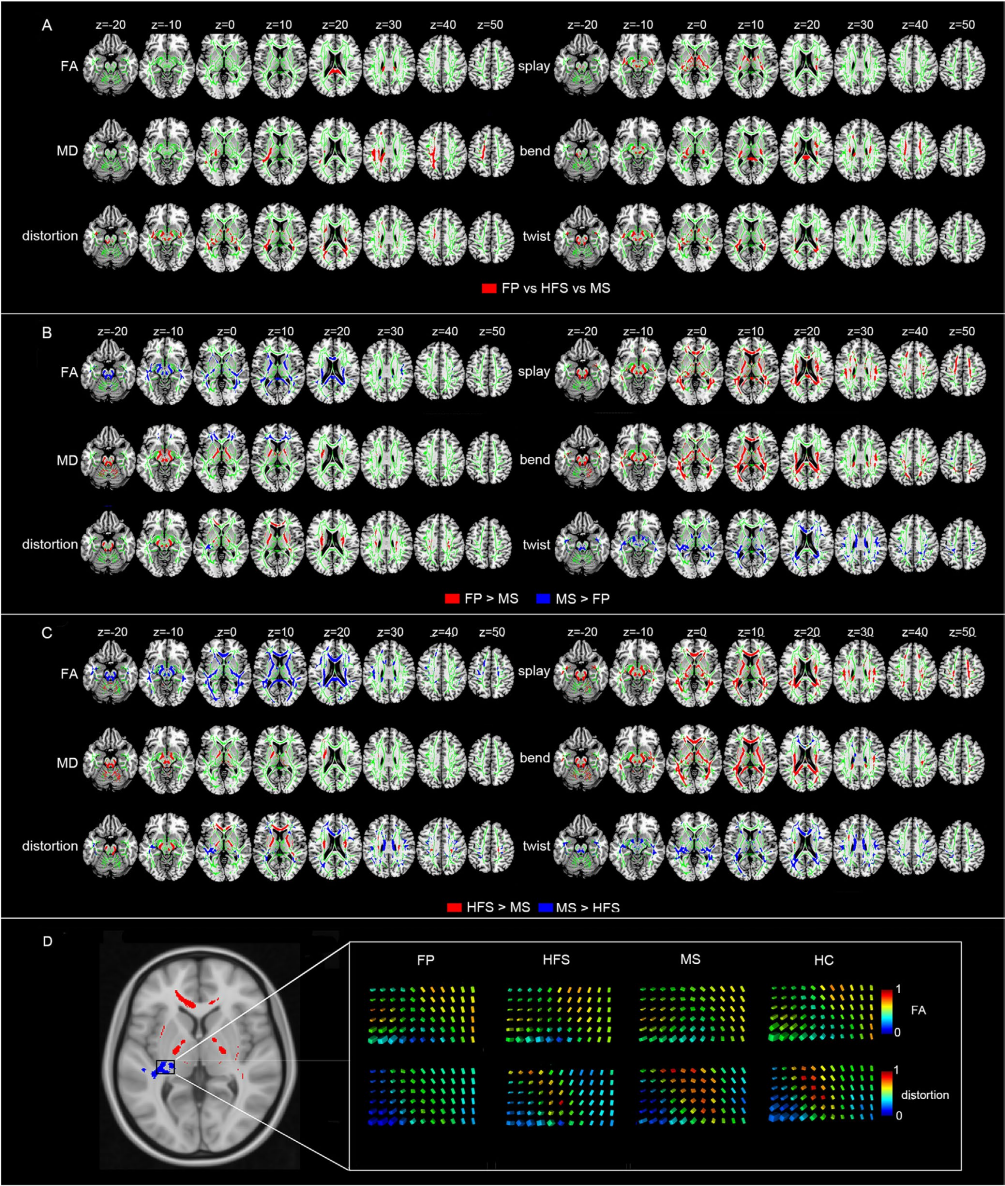
Microstructural changes in the orientation (i.e., splay, bend, twist, total distortion) and integrity (i.e., FA, MD) among three different subtypes of facial dyskinesias across the whole brain white matter. (A) Changes in the orientation and integrity of one‐way ANOVA on three different subtypes of facial dyskinesias. (B) Changes in the orientation and integrity in the FP patients compared to MS patients. (C) Changes in the orientation and integrity in the HFS patients compared to MS patients. (D) Sketch map of the significant differences of the total distortion index in the tensor field in FP compared to MS. We visualized the tensor field by coloring the glyphs using the total distortion index and FA in one of the significant different regions (white box) in FP, HFS, MS, and HC. We can visually observe the significant differences of the total distortion in the tensor field (lower box) in the given region where the FA (upper box) is visually similar in FP, HFS, MS, and HC. Significant voxels (*p* < 0.05, corrected for multiple comparisons) were thickened by using the TBSS fill function into local tracts (red/blue) and overlaid on the white matter skeleton (green). A complete listing of all significant white matter tracts is provided in Tables S5–S7.

The one‐way ANOVA revealed significantly different FA in corpus callosum, MD in superior longitudinal fasciculus, internal capsule, and corona radiata, and DFA metrics values in corona radiata, internal capsule, thalamic radiata, cerebellar peduncle, corpus callosum, fornix, and external capsule (Figure 3A) and Table S5. The results of post hoc analyses in significant clusters were summarized in Figures S1 and S2.

Moreover, the results of t‐tests demonstrated significant differences between FP and MS patients, as well as between HFS and MS patients. Specifically, compared with MS patients, FP patients showed significantly decreased FA, MD, and DFA metrics (total distortion, bend, and twist) in the corpus callosum, superior longitudinal fasciculus, and internal capsule. Additionally, decreased MD was found in the cerebellar peduncle and corona radiata, and twist in the thalamic radiata. Conversely, FP patients showed significantly increased MD in cerebellar peduncle and internal capsule, and increased DFA metrics (total distortion, splay, bend) in corona radiata, internal capsule, corpus callosum, superior longitudinal fasciculus, and thalamic radiata compared with MS patients (Figure 3B and Table S6).

Compared with MS patients, HFS patients showed significant decreases in FA and DFA metrics (total distortion, bend, twist) in corpus callosum, thalamic radiata, superior longitudinal fasciculus, and corona radiata. Decreased FA in the cerebellar peduncle and DFA in the internal capsule and fornix were also observed. Conversely, HFS patients showed significantly increased FA/MD and DFA metrics (total distortion, splay, bend, and twist) in cerebellar peduncle, internal capsule, and corona radiata. Increased DFA metrics were also observed in the external capsule, corpus callosum, and superior longitudinal fasciculus (Figure 3C and Table S7). No difference was found between FP patients and HFS patients.

As depicted in Figure 3D, the blue region indicates distinct geometric microstructural changes among FP, HFS, MS, and HC groups, while FA visually appears similar.

### 3.4 Correlation Analysis Between DFA and Clinical Data

**FIGURE 4:**
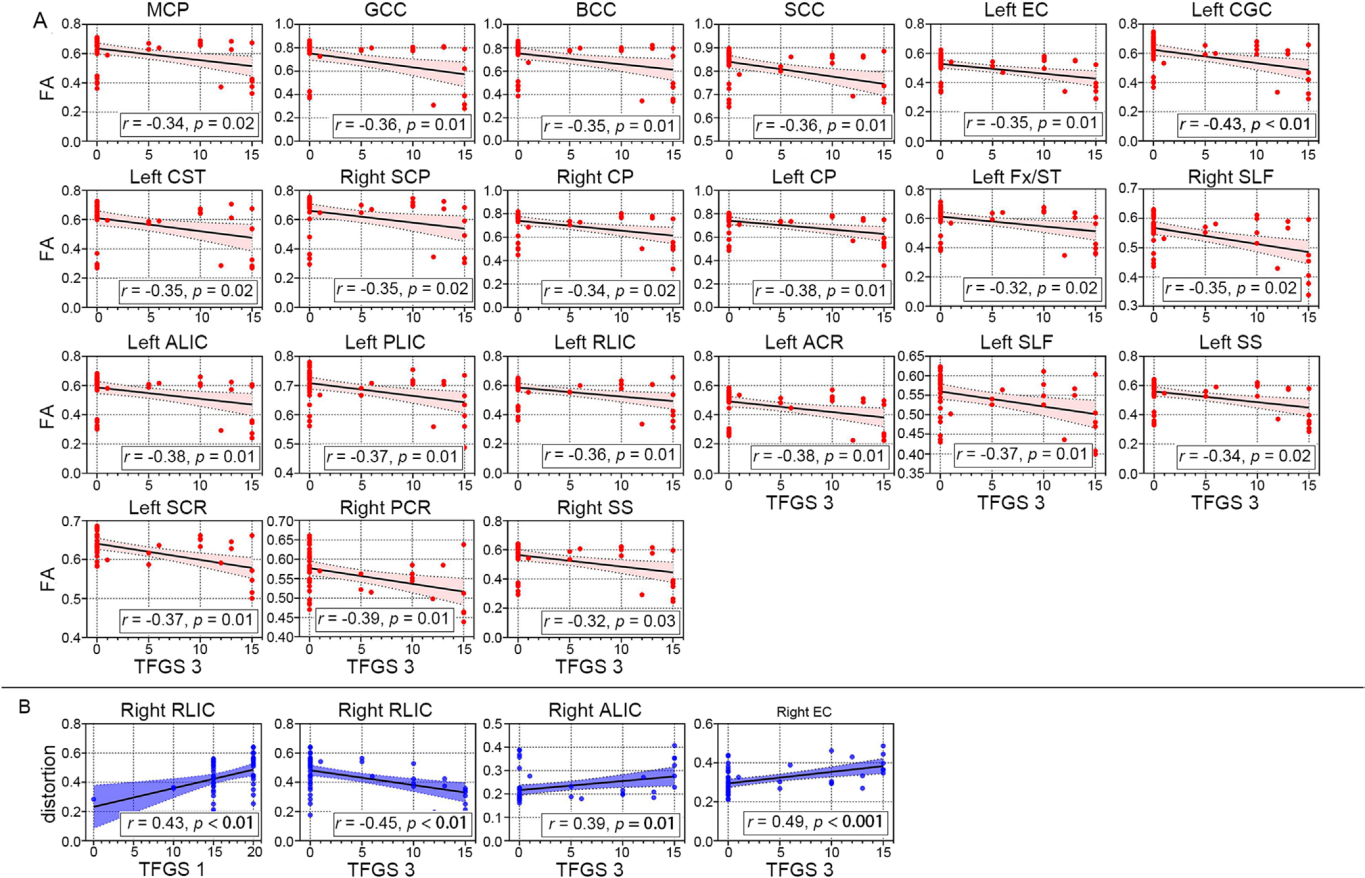
ROI‐wise scatterplot and linear correlation between FA indices and clinical variables, and between DFA indices and clinical variables in FP patients and MS patients. (A) Correlation between FA of ROIs and TFGS_3 scores in FP patients. Light red area indicated 95% CIs along the fitted line (black line). (B) Correlation between distortion of ROIs and TFGS_3 scores in FP patients. Light blue area indicated 95% CIs along the fitted line (blue line). Orange and blue area indicated 95% CIs along the regression line (black line). The corresponding correlation coefficients and corrected *P* values were denoted in the bottom right corner.

The results of correlation analysis were shown in Figure 4. Specifically, in FP patients, we found a significant negative correlation between TFGS_3 and FA within the cerebellar peduncle, callosum corpus, internal and external capsule, cingulum, corticospinal tract, superior longitudinal fasciculus, sagittal stratum, and fornix (Figure 4A). Additionally, a significant positive correlation was found between TFGS_1 and total distortion within the right retrolenticular part of the internal capsule. Moreover, a positive correlation was observed between TFGS_3 and total distortion in the right anterior limb of the internal and external capsule. Furthermore, TFGS_3 negatively correlated with distortion in the right retrolenticular part of the internal capsule (Figure 4B). The correlation results between the other three DFA metrics and TFGS_3 are shown in Figure S3.

### 3.5 Group Differences of DTI Metrics Between HC_1 and HC_2

The results of group comparisons between HC_1 and HC_2 revealed significant differences in FA, MD, and DFA metrics, primarily located in the corpus callosum, corona radiata, and superior longitudinal fasciculus Figure S4 and Table S8. These findings are consistent with the well‐documented effects of aging on white matter microstructure, given the significant age difference between the younger HC_1 group and the older HC_2 group.

### 3.6 Classification of FP, HFS and MS

**FIGURE 5:**
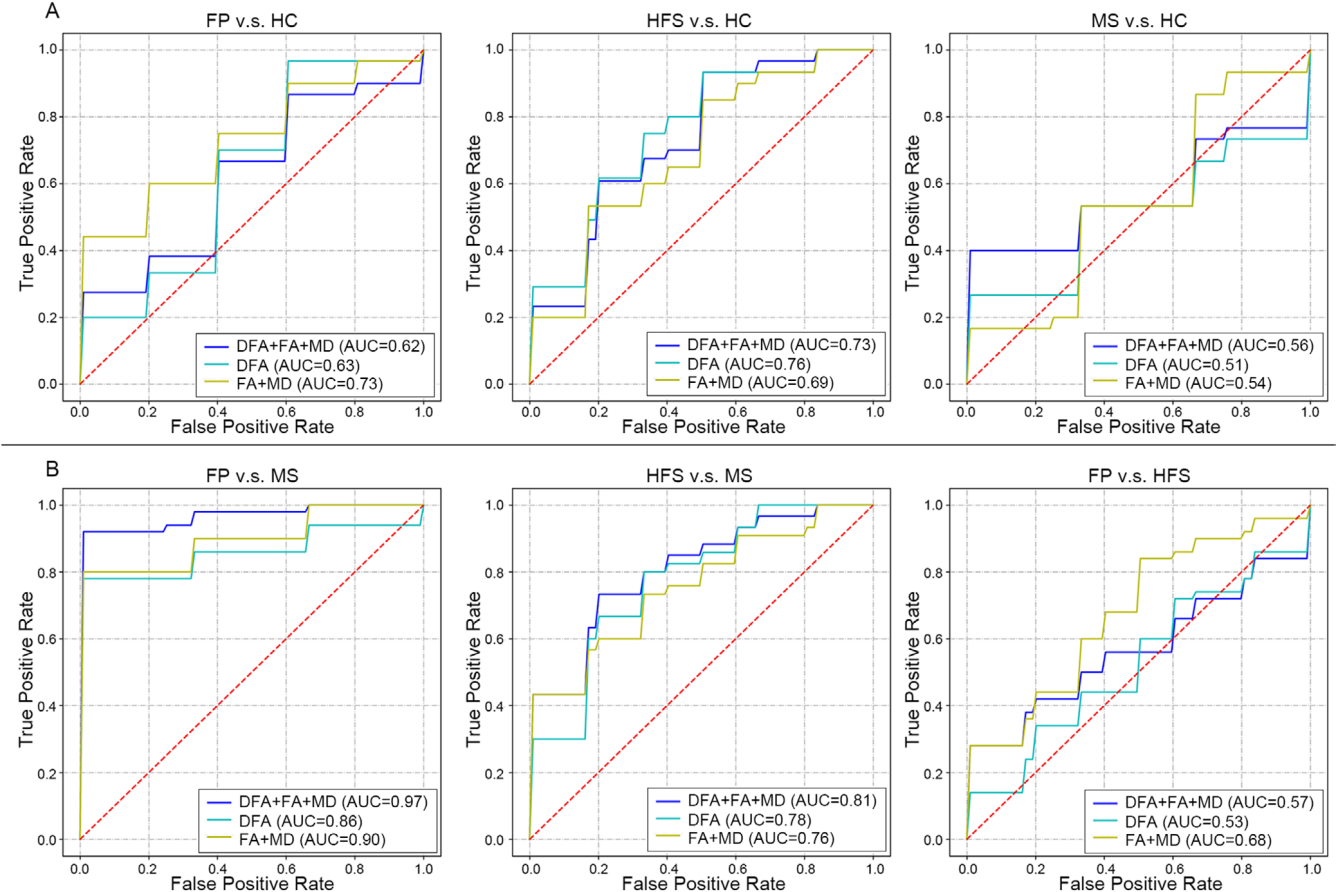
Area under the receiver operating characteristic (ROC) curve values with classification to distinguish participants with facial dyskinesias from healthy control subjects and distinguish among three different subtypes of facial dyskinesias. (A) ROC curves for distinguishing FP and HC_1, ROC curves for distinguishing HFS and HC_1 and ROC curves for distinguishing MS and HC_2. (B) ROC curves for distinguishing FP and MS, ROC curves for distinguishing HFS and MS and ROC curves for distinguishing FP and HFS. All feature matrices of all three models (model1: DFA + FA + MD; model2: DFA; model3: FA + MD) were included for every ROC analysis, respectively. Blue line denoted feature model containing DFA, FA, and MD indices. Green line denoted feature model containing DFA indices. Yellow line denoted feature model FA and MD indices. False‐positive rate represented 1 minus specificity. True‐positive rate represented sensitivity. A complete listing of all classification performances is provided in Table S9.

The results of classification between facial dyskinesias and HC (Figure 5A and Table S9) revealed that the AutoGluon classifier with FA combined MD can moderate classification performance for FP patients and HC (AUC = 0.7313). However, the classifier with DFA metrics alone performed worse than the FA combined with MD classifier for classifying FP patients and HC (AUC = 0.6333). Combined FA, MD, and DFA metrics only led to a limited worsening in classifying FP patients and HC (AUC = 0.6183).

Conversely, the classifiers with FA + MD + DFA (AUC = 0.7328) and DFA (AUC = 0.755) showed better classification performance than the classifier with FA + MD (AUC = 0.6944) in differentiating HFS patients and HC. However, all three classifiers did not perform well in classifying MS patients and HC, with AUC < 0.6.

The results of classification between FP and MS patients, between HFS and MS patients, and between FP and HFS patients were shown in Figure 4B. The classifiers utilizing DFA metrics and FA + MD can effectively classify FP and MS patients with AUC > 0.85. Combining FA/MD and DFA metrics, the classifier achieved the highest performance (AUC = 0.9683) in classifying FP and MS patients. Besides, the tree classifiers showed moderate discriminative ability (AUC > 0.75) in differentiating HFS and MS patients. However, the classification capability of those three classifiers shows more limited discriminative power (AUC < 0.7) in differentiating FP and HFS patients.

Additionally, the results demonstrate that both individual classification models and the AutoGluon stacked ensemble performed significantly better in distinguishing between FP vs. MS and HFS vs. MS than in classifying FP vs. HFS Table S10.

## 4 Discussion

Facial paralysis (FP), hemifacial spasm (HFS), and Meige's syndrome (MS) exhibit diverse clinical manifestations, making their differentiation challenging in clinical practice. Furthermore, the underlying neurological mechanisms remain poorly understood. In this study, we aimed to comprehensively examine white matter abnormalities by analyzing various indices, including fractional anisotropy (FA), mean diffusivity (MD), and directional fractional anisotropy (DFA) across the entire white matter skeleton. Additionally, we explored the relationship between altered white matter indices and clinical symptoms in FP, HFS, and MS. Furthermore, we assessed the differential diagnostic ability of DFA metrics compared to traditional FA/MD values. Our main findings are as follows: (i) FP and HFS patients exhibited widespread changes in FA/MD and DFA metrics across the entire white matter compared to HC, whereas MS patients showed significant alterations only in DFA metrics; (ii) DFA metrics revealed a greater number of significant clusters in group comparisons between FP, HFS, and MS patients compared to traditional FA/MD values; (iii) Both FA and DFA values exhibited a strong correlation with the severity of facial movement disorders in FP patients; (iv) The combination of conventional FA and MD metrics with DFA metrics facilitated the diagnostic differentiation of FP and HFS from MS. These findings underscore the potential of advanced imaging techniques such as DFA in enhancing the diagnosis and characterization of FP, HFS, and MS, while shedding light on the underlying pathophysiology.

Although HFS and FP are neuromuscular diseases characterized by abnormal function of the facial nerve in the cerebellar pontine cistern, the reorganization of the central nervous system is also considered a potential pathological mechanism for facial diseases. This hypothesis is supported by strong evidence from previous structural and functional MRI studies [5, 11, 12, 22, 23, 24]. However, comprehensive knowledge regarding alterations in white matter microstructure in facial movement disorders is limited. Traditional DTI‐derived metrics, such as FA and MD, have typically been used to assess myelin integrity in white matter, and lack of description of the orientational properties of microstructure in the white matter fibers in a spatial neighborhood. The introduction of the DFA metric enables us to thoroughly investigate the geometric characteristics of white matter in facial disorders.

Specifically, our study revealed widespread changes in FA, MD, and DFA metrics in various brain regions of both HFS and FP patients. These changes were predominantly characterized by lower FA, increased twist, and higher MD distortion, splay, and bend in regions such as the corpus callosum, thalamic radiation, internal capsule, cerebellar peduncle, corona radiata, and fronto‐occipital fasciculus. These white matter tracts connect gray matter regions known to be involved in the multifaceted aspects of facial motor function, face perception, and emotion modulation, which is consistent with previous research findings [11]. The intersection between fibers originating from the brainstem and the significant clusters of the distortion metric is shown in Figure S5. The majority of these fibers are associated with motor efferent fibers and the facial nucleus. Furthermore, there is a clear overlap between the significant clusters of the distortion metric and these fibers, particularly in patients HFS. These findings suggest that geometric alterations in white matter may underlie the pathophysiology of facial motor disorders. Additionally, previous studies on functional and structural networks have indicated abnormal reorganization in the rich‐club in facial synkinesis [5], and our findings may provide structural evidence for the alterations in the topology of the brain network.

Also, it is important to note that the two healthy control groups (HC_1 and HC_2) themselves showed differences in white matter metrics, attributable to their age disparity. This observation underscores the necessity and validity of our study design, which used age‐matched control groups (HC_1 for FP/HFS; HC_2 for MS) to ensure that the microstructural alterations identified in patient groups are related to the disease state rather than confounding age effects.

In addition, we observed a strong association between FA and DFA values and the synkinesis score (TFGS_3) in patients with facial palsy. Facial synkinesis is a notable complication of facial palsy [25]. Recent evidence suggests that facial synkinesis results from extensive cortical reorganization; previous series of studies revealed significant alterations in neural plasticity [26]. Our results suggested that geometric microstructural DFA metrics and traditional metrics can serve as potential biomarkers for monitoring the disease progression of synkinesis in FP.

However, the presence of significant clusters in FA/MD metrics was much less common in patients with multiple sclerosis (MS), indicating that the pathogenesis and etiology of MS may differ from that of facial palsy (FP) and hemifacial spasm (HFS). Previous studies have suggested that the dysfunction of the basal ganglia and the lack of inhibition at various levels in the central nervous system contribute to hyperactivity in MS patients [27, 28]. Compared to conventional DTI metrics (FA/MD), MS exhibited significant alterations in white matter geometric features in the corpus callosum and corona radiate, indicating that DFA metrics are more sensitive imaging biomarkers for MS.

To further understand the underlying mechanisms of these three facial movement disorders, additional TBSS analyses were conducted. DFA metrics revealed more extensive changes in white matter regions throughout the entire brain, particularly in the thalamic radiation and internal capsule. While both FP and HFS exhibited widespread alterations in FA/MD and DFA metrics compared to MS, there were no significant clusters in any DFA or FA/MD metrics between FP and HFS. These significant tracts are involved in the cortico‐basal ganglia‐cortical and cortico‐cerebello‐cortical circuits, which strongly influence the abnormal electrical impulses of the facial nerve or facial nucleus [29]. Our findings suggested that the observed differences between MS, FP, and HFS may be primarily attributed to geometric microstructural changes in the white matter, rather than alterations in white matter integrity. These microstructural changes can disrupt the normal communication between different brain regions. A previous study also reported structural dysconnectivity between the thalamus, occipital cortex, and pre‐motor cortex in MS, which could contribute to abnormal sensory processing, particularly in the visual domain, as well as dysfunctional ascending motor control [30]. Our results provide strong evidence supporting that geometric changes of thalamic radiation can be the pathological explanation for the thalamic connectivity theory in MS.

The results of classifications among facial movement disorders also proved the different pathological mechanisms between MS, FP, and HFS. The DFA metrics combining with FA/MD can effectively differentiate MS patients from other facial movement disorders, especially the classification between FP and MS with the highest AUC. However, the classification capability of FA/MD, DFA, and their combinations showed weak ability in differentiating FP and HFS patients. The AUC values enabled us to confirm that combing conventional FA/MD and DFA metrics is useful to differentiate MS from FP and HFS. Also, the DFA metrics assisted the diagnostic performance in FP and HFS from healthy controls. Furthermore, the performance of AUC indicated similar CNS pathological mechanisms in both FP and HFS, which was different from MS patients.

### 4.1 Limitations

In this study, we collected brain imaging data from 50 patients with FP, 57 patients with HFS, and 31 patients with MS. Considering the challenges in acquiring imaging data for patients with facial palsy and its subtypes—such as the fact that treatment is often conducted in community hospitals where clinical imaging is not routinely performed—our sample size is still relatively larger compared to other studies focusing on brain imaging in facial palsy and its subtypes [31, 32, 33]. Given the current lack of extensive brain imaging research in facial palsy and its subtypes, we hope that future studies will provide opportunities for large‐scale, comprehensive analyses using big data cohorts to further explore these conditions. Lastly, while our machine learning approach demonstrated the potential of DFA metrics for differentiation, particularly between FP/HFS and MS, the classification performance could be further validated and potentially improved in future studies. This could be achieved by employing statistical significance testing for model performance (e.g., permutation tests) and by incorporating a broader set of features, such as morphometric data, functional connectivity measures, or clinical variables, to build more powerful and clinically translatable diagnostic models.

## 5 Conclusion

In conclusion, our study represents a pioneering effort in utilizing both traditional and DFA metrics to unveil a comprehensive brain‐wide pattern of microstructural alterations. These changes, reflective of myelin integrity and directional organization within white matter tracts, were observed in patients with HFS, FP, and MS. Furthermore, we elucidated the association between these microstructural variations and the diminished synkinesis performance.

Our findings highlight the possibility of white matter geometric structural features as potential biomarkers for monitoring the disease progression and as a diagnostic index to differentiate among HFS, FP, and MS and may offer novel insights into the underlying pathological mechanisms.

## Author Contributions

Conception and design: Hua Zhu, Aocai Yang, Jian Cheng, and Guolin Ma; Administrative support: Jian Cheng and Guolin Ma; Provision of study materials or patients: All authors; Collection and assembly of data: Aocai Yang, Hua Zhu, Bing Liu, Jixin Luan, Manxi Xu, Kuan Lv, and Pianpian Hu; Data analysis and interpretation: Hua Zhu, Aocai Yang, Amir Shmuel, Haogang Zhu, Zhen Yuan, Jian Cheng, and Guolin Ma; Manuscript writing: Hua Zhu and Aocai Yang; Manuscript editing: Hua Zhu, Aocai Yang, Amir Shmuel, Haogang Zhu, Zhen Yuan, Jian Cheng, and Guolin Ma; Final approval of manuscript: All authors.

## Funding

This study was partially supported by Beijing Natural Science Foundation (Grant No. 4252004, L242038), National Key Research and Development Program Intergovernmental Key Project of China (Grant No. 2024YFE0100900), Chinese Institutes for Medical Research Beijing (Grant No. CX25YQ11), the National Natural Science Foundation of China (61971017, 82271953, U25A20136), 2030‐“Brain Science and Brain‐Like Intelligence Technology” Project of China (2022ZD0213300), Open Research Fund of the State Key Laboratory of Cognitive Neuroscience and Learning (CNLZD2303).

## Ethics Statement

This study was approved by the Ethics Committee of the China‐Japan Friendship Hospital (Approval ID: 2020‐KY‐181) and conducted in accordance with the Declaration of Helsinki. All the participants and/or their relatives were informed about this study and provided their written informed consent.

## Consent

The participants were informed and they agreed to participate in this study.

## Conflicts of Interest

The authors declare no conflicts of interest.

## Supporting information


**Figure S1:** ROI‐wise one‐way ANOVA results of FA, MD, and distortion among three subtypes of facial dyskinesia patients. * denotes *p* < 0.05. ** denotes *p* < 0.01. *** denotes *p* < 0.001.
**Figure S2:** ROI‐wise one‐way ANOVA results of FA, MD, and distortion among three subtypes of facial dyskinesia patients. * denotes *p* < 0.05. ** denotes *p* < 0.01. *** denotes *p* < 0.001.
**Figure S3:** ROI‐wise scatterplot and linear correlation between DFA metrics and clinical variables in FP patients.
**Figure S4:** Microstructural changes in the orientation and integrity between HC_1 and HC_2 across the whole brain white matter.
**Figure S5:** Schematic representation of fibers originating from the brainstem and the clusters of distortion index with significant differences compared to HC in various patient groups. (A) FP patients. (B) HFS patients. (C) MS patients. (D) The clusters of distortion index with significant changes identified through a one‐way ANOVA across all patient groups.
Table S1: List of abbreviations for white matter bundles.
**Table S2:** The results of TBSS between FP and HC in DTI metrics.
**Table S3:** The results of TBSS between HFS and HC in DTI metrics.
**Table S4:** The results of TBSS between MS patients and HC in DTI metrics.
**Table S5:** The results of TBSS of one‐way ANOVA among three facial diseases in DTI metrics.
**Table S6:** The results of TBSS between FP and MS patients in DTI metrics.
**Table S7:** The results of TBSS between HFS and MS patients DTI metrics.
**Table S8:** The Results of TBSS between HC_1 and HC_2 patients in DTI metrics.
**Table S9**: The Results of classification using AutoGluon.
Table S10: Results of classification using SVM, Logistic Regression, and Random Forests.

## Data Availability

The data that support the findings of this study are available on request from the corresponding author. The data are not publicly available due to privacy or ethical restrictions.
