# Supplementary material for: Geometric Microstructural Characteristics of White Matter Differentiate Patients With Facial Dyskinesias and Palsy
Source: CNS Neurosci Ther. 2026 Jan 28;32(2):e70733. doi: 10.1002/cns.70733 (PMC12848538; doi:10.1002/cns.70733)
Supplement: Supplementary file 1 — Figure S1: ROI‐wise one‐way ANOVA results of FA, MD, and distortion among three subtypes of facial dyskinesia patients. * denotes p < 0.05. ** denotes p < 0.01. *** denotes p < 0.001. Figure S2: ROI‐wise one‐way ANOVA results of FA, MD, and distortion among three subtypes of facial dyskinesia patients. * denotes p < 0.05. ** denotes p < 0.01. *** denotes p < 0.001. Figure S3: ROI‐wise scatterplot and linear correlation between DFA metrics and clinical variables in FP patients. Figure S4: Microstructural changes in the orientation and integrity between HC_1 and HC_2 across the whole brain white matter. Figure S5: Schematic representation of fibers originating from the brainstem and the clusters of distortion index with significant differences compared to HC in various patient groups. (A) FP patients. (B) HFS patients. (C) MS patients. (D) The clusters of distortion index with significant changes identified through a one‐way ANOVA across all patient groups. Table S1: List of abbreviations for white matter bundles. Table S2: The results of TBSS between FP and HC in DTI metrics. Table S3: The results of TBSS between HFS and HC in DTI metrics. Table S4: The results of TBSS between MS patients and HC in DTI metrics. Table S5: The results of TBSS of one‐way ANOVA among three facial diseases in DTI metrics. Table S6: The results of TBSS between FP and MS patients in DTI metrics. Table S7: The results of TBSS between HFS and MS patients DTI metrics. Table S8: The Results of TBSS between HC_1 and HC_2 patients in DTI metrics. Table S9: The Results of classification using AutoGluon. Table S10: Results of classification using SVM, Logistic Regression, and Random Forests. [file CNS-32-e70733-s001.docx]

**Supplemental Figure**

**
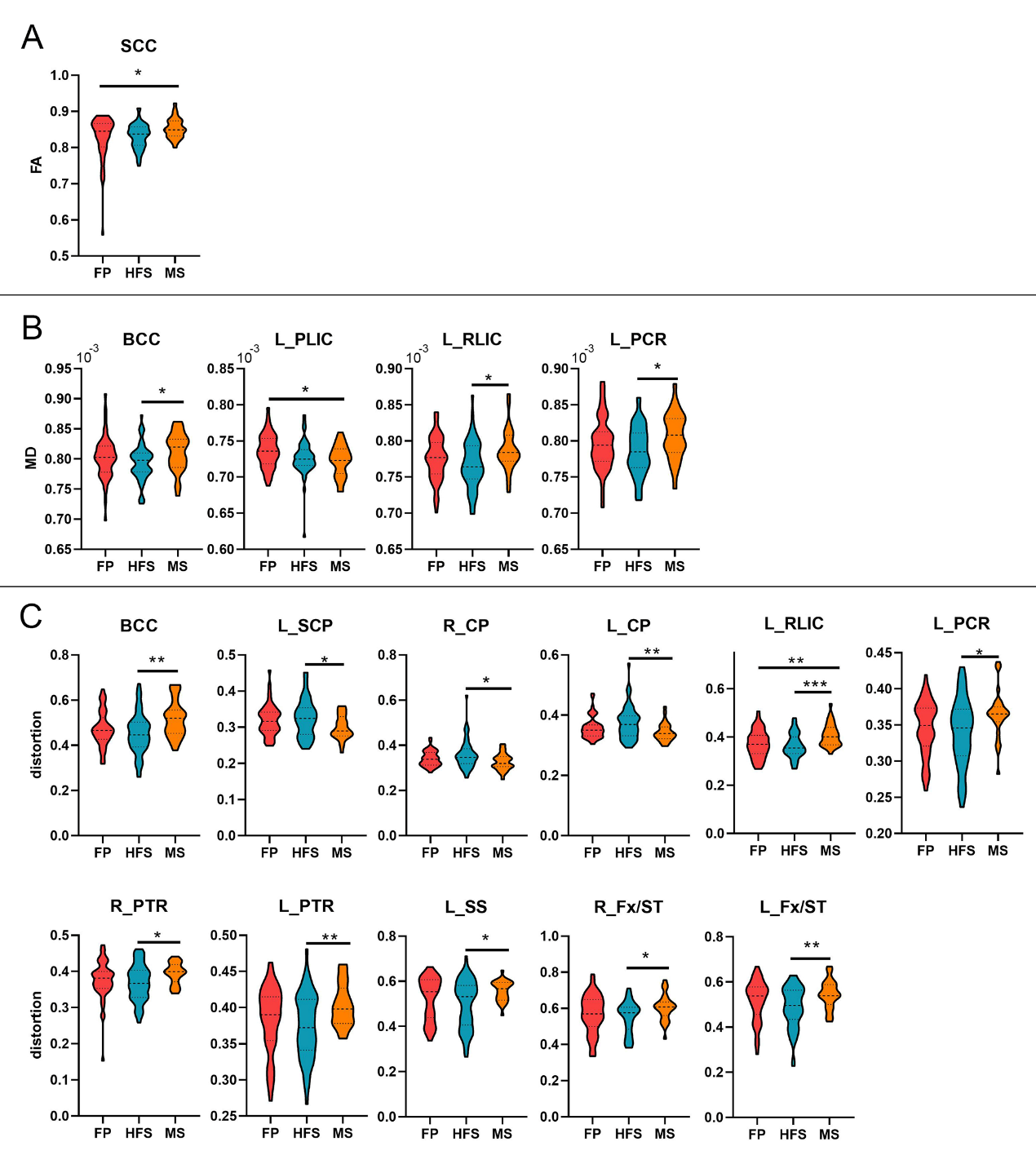
**

**Figure S1** ROI-wise one-way ANOVA results of FA, MD and distortion among three subtypes of facial dyskinesia patients. * denotes p < 0.05. ** denotes p < 0.01. *** denotes p < 0.001.


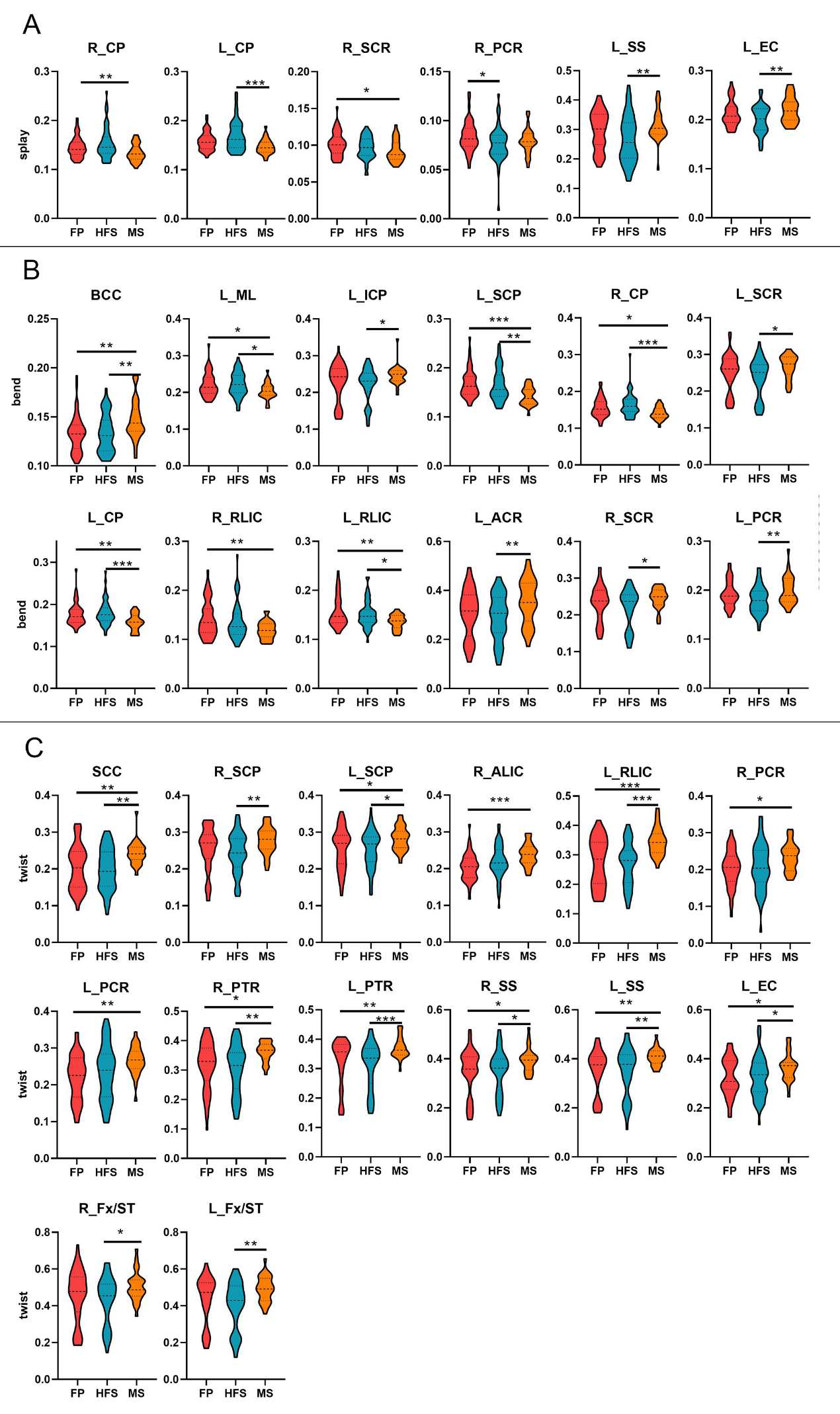


**Figure S2** ROI-wise one-way ANOVA results of FA, MD and distortion among three subtypes of facial dyskinesia patients. * denotes p < 0.05. ** denotes p < 0.01. *** denotes p < 0.001.


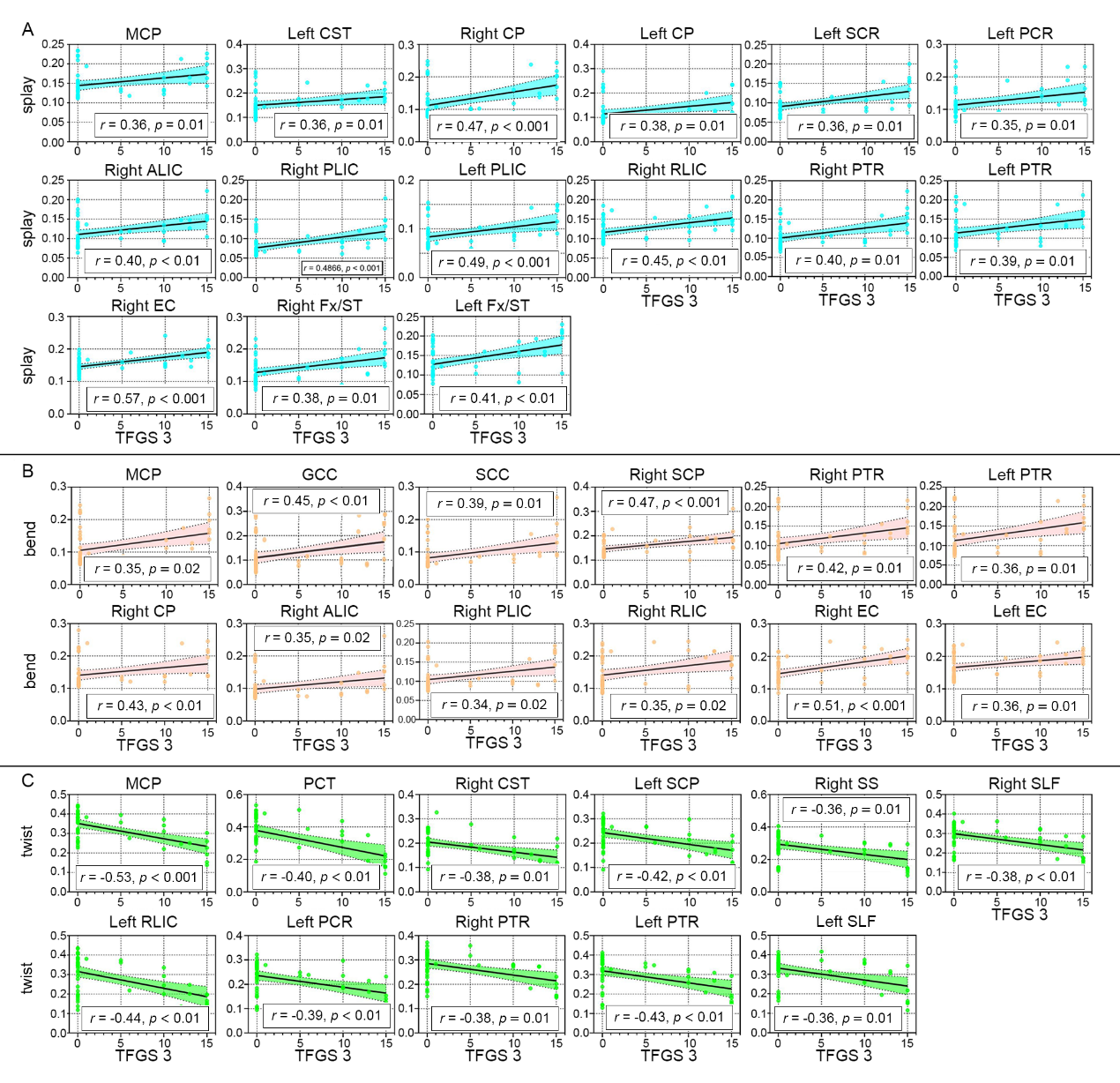


**Figure S3** ROI-wise scatterplot and linear correlation between DFA metrics and clinical variables in FP patients.


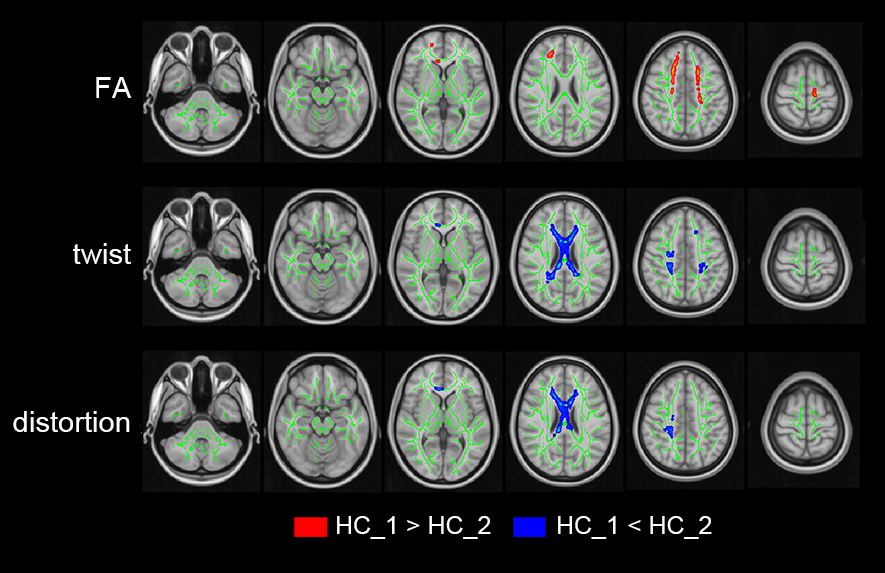


**Figure S4** Microstructural changes in the orientation and integrity between HC_1 and HC_2 across the whole brain white matter.


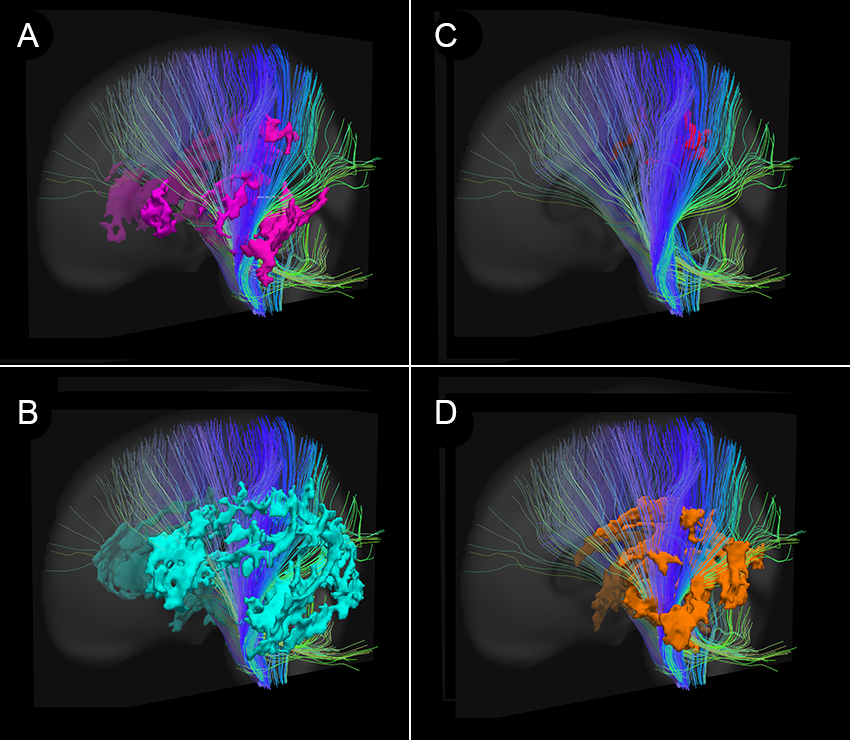


**Figure S5** Schematic representation of fibers originating from the brainstem and the clusters of distortion index with significant differences compared to HC in various patient groups. (A) FP patients. (B) HFS patients. (C) MS patients. (D) The clusters of distortion index with significant changes identified through a one-way ANOVA across all patient groups.

**Supplemental Tables**

Table S1: List of abbreviations for white matter bundles

| Abbreviations | Full name of WM bundles |
| --- | --- |
| MCP | Middle cerebellar peduncle |
| PCT | Pontine crossing tract |
| GCC | Genu of corpus callosum |
| BCC | Body of corpus callosum |
| SCC | Splenium of corpus callosum |
| Fx | Fornix (column and body of fornix) |
| CST | Corticospinal tract |
| ML | Medial lemniscus |
| ICP | Inferior cerebellar peduncle |
| SCP | Superior cerebellar peduncle |
| CP | Cerebral peduncle |
| ALIC | Anterior limb of internal capsule |
| PLIC | Posterior limb of internal capsule |
| RLIC | Retrolenticular part of internal capsule |
| ACR | Anterior corona radiata |
| SCR | Superior corona radiata |
| PCR | Posterior corona radiata |
| PTR | Posterior thalamic radiation |
| SS | Sagittal stratum |
| EC | External capsule |
| CGC | Cingulum (cingulate gyrus) |
| CGH | Cingulum (hippocampus) |
| Fx/ST | Fornix/stria terminalis |
| SLF | Superior longitudinal fasciculus |
| SFOF | Superior fronto-occipital fasciculus |
| IFOF | Inferior fronto-occipital fasciculus |
| UF | Uncinate fasciculus |
| TAP | Tapetum |

Table S2: The results of TBSS between FP and HC in DTI metrics.

| Contrast group | Cluster index | Cluster size | *P_perm_* value | Peak MNI  coordinates (mm) | | | White matter location  (JHU-81 Atlas) | |
| --- | --- | --- | --- | --- | --- | --- | --- | --- |
|  |  |  |  | X | Y | Z | Regions | Voxel number |
| HC > FP | | | | | | | | |
| FA | 1 | 28868 | 0.02 | 133 | 113 | 49 | SCC | 1764 |
|  |  |  |  |  |  |  | MCP | 871 |
|  |  |  |  |  |  |  | L_PTR | 800 |
|  |  |  |  |  |  |  | R_PTR | 743 |
|  |  |  |  |  |  |  | L_ALIC | 737 |
|  | 2 | 1444 | 0.042 | 88 | 151 | 72 | GCC | 714 |
|  |  |  |  |  |  |  | BCC | 679 |
| distortion | 1 | 1212 | 0.026 | 138 | 98 | 74 | L_RLIC | 239 |
|  |  |  |  |  |  |  | L_Fx/ST | 74 |
|  |  |  |  |  |  |  | L_SS | 69 |
|  | 2 | 299 | 0.026 | 121 | 107 | 113 | L_SLF | 299 |
|  | 3 | 232 | 0.036 | 53 | 150 | 89 | R_IFOF | 232 |
| twist | 1 | 18294 | 0.002 | 131 | 117 | 45 | R_PTR | 818 |
|  |  |  |  |  |  |  | L_RLIC | 654 |
|  |  |  |  |  |  |  | L_SLF | 515 |
|  |  |  |  |  |  |  | SCC | 514 |
|  |  |  |  |  |  |  | L_PTR | 466 |
|  | 2 | 2412 | 0.022 | 61 | 140 | 181 | R_SLF | 252 |
|  |  |  |  |  |  |  | R_ACR | 217 |
|  |  |  |  |  |  |  | R_ALIC | 111 |
|  |  |  |  |  |  |  | R_EC | 76 |
|  |  |  |  |  |  |  | R_SFOF | 69 |
|  | 3 | 1654 | 0.016 | 61 | 84 | 109 | R_SLF | 127 |
|  | 4 | 1158 | 0.014 | 95 | 92 | 38 | MCP | 224 |
|  |  |  |  |  |  |  | PCT | 208 |
|  |  |  |  |  |  |  | R_CST | 89 |
|  |  |  |  |  |  |  | L_SCP | 71 |
|  | 5 | 206 | 0.044 | 83 | 107 | 58 | R_ATR | 206 |
| FP > HC | | | | | | | | |
| MD | 1 | 8045 | 0.028 | 119 | 119 | 88 | MCP | 868 |
|  |  |  |  |  |  |  | L_SCR | 681 |
|  |  |  |  |  |  |  | L_PLIC | 451 |
|  |  |  |  |  |  |  | L_ALIC | 282 |
|  |  |  |  |  |  |  | L_EC | 256 |
| distortion | 1 | 2836 | 0.002 | 77 | 104 | 53 | R_SCR | 619 |
|  |  |  |  |  |  |  | R_PLIC | 563 |
|  |  |  |  |  |  |  | R_CP | 414 |
|  |  |  |  |  |  |  | R_EC | 257 |
|  |  |  |  |  |  |  | R_ALIC | 247 |
|  | 2 | 1511 | 0.004 | 94 | 155 | 80 | GCC | 858 |
|  |  |  |  |  |  |  | R_ACR | 236 |
|  |  |  |  |  |  |  | L_ACR | 268 |
|  | 3 | 1402 | 0.008 | 110 | 118 | 79 | L_ALIC | 196 |
|  |  |  |  |  |  |  | L_PLIC | 530 |
|  |  |  |  |  |  |  | L_SCR | 448 |
|  |  |  |  |  |  |  | L_EC | 152 |
|  | 4 | 234 | 0.038 | 103 | 105 | 54 | L_CP | 230 |
|  | 5 | 154 | 0.04 | 98 | 100 | 63 | L_ATR | 154 |
| splay | 1 | 23114 | 0.002 | 87 | 89 | 29 | SCC | 1348 |
|  |  |  |  |  |  |  | BCC | 1141 |
|  |  |  |  |  |  |  | GCC | 1072 |
|  |  |  |  |  |  |  | L_PTR | 842 |
|  |  |  |  |  |  |  | R_SCR | 786 |
|  | 2 | 1134 | 0.02 | 110 | 109 | 120 | L_SCR | 78 |
| bend | 1 | 16757 | 0.002 | 83 | 87 | 39 | SCC | 1951 |
|  |  |  |  |  |  |  | L_PTR | 1057 |
|  |  |  |  |  |  |  | R_PTR | 969 |
|  |  |  |  |  |  |  | L_PLIC | 651 |
|  |  |  |  |  |  |  | R_PLIC | 612 |
|  | 2 | 991 | 0.022 | 100 | 156 | 71 | GCC | 744 |
|  |  |  |  |  |  |  | R_ACR | 115 |
|  |  |  |  |  |  |  | L_ACR | 104 |
|  | 3 | 238 | 0.038 | 72 | 84 | 35 | MCP | 238 |
|  | 4 | 219 | 0.04 | 62 | 111 | 104 | R_SCR | 134 |

Note: To briefly present the results, the top five white matter fiber regions with the highest number of voxels in each cluster were shown in the table. L: Left; R: Right.

Table S3: The results of TBSS between HFS and HC in DTI metrics.

| Contrast group | Cluster index | Cluster size | *P_perm_* value | Peak MNI  coordinates (mm) | | | White matter location  (JHU-81 Atlas) | |
| --- | --- | --- | --- | --- | --- | --- | --- | --- |
|  |  |  |  | X | Y | Z | Regions | Voxel number |
| HC > HFS | | | | | | | | |
| FA | 1 | 51917 | 0.002 | 87 | 89 | 22 | SCC | 1988 |
|  |  |  |  |  |  |  | GCC | 1745 |
|  |  |  |  |  |  |  | MCP | 1692 |
|  |  |  |  |  |  |  | BCC | 1376 |
|  |  |  |  |  |  |  | L_ACR | 1134 |
| distortion | 1 | 5479 | 0.004 | 130 | 117 | 50 | L_RLIC | 380 |
|  |  |  |  |  |  |  | L_SLF | 330 |
|  |  |  |  |  |  |  | L_Fx/ST | 203 |
|  |  |  |  |  |  |  | L_SS | 126 |
|  |  |  |  |  |  |  | L_PTR | 122 |
|  | 2 | 4251 | 0.006 | 42 | 119 | 96 | R_SLF | 337 |
|  |  |  |  |  |  |  | R_ACR | 208 |
|  |  |  |  |  |  |  | R_EC | 135 |
|  |  |  |  |  |  |  | R_SCR | 77 |
|  | 3 | 2278 | 0.006 | 62 | 103 | 66 | R_SS | 288 |
|  |  |  |  |  |  |  | R_PTR | 189 |
|  |  |  |  |  |  |  | R_Fx/ST | 156 |
|  |  |  |  |  |  |  | R_RLIC | 144 |
|  |  |  |  |  |  |  | R_EC | 93 |
|  | 4 | 2024 | 0.008 | 109 | 161 | 96 | BCC | 408 |
|  |  |  |  |  |  |  | L_ACR | 346 |
|  |  |  |  |  |  |  | L_SCR | 69 |
|  |  |  |  |  |  |  | GCC | 67 |
|  |  |  |  |  |  |  | L_ALIC | 53 |
|  | 5 | 1106 | 0.018 | 116 | 63 | 86 | L_PCR | 157 |
|  |  |  |  |  |  |  | L_PTR | 72 |
|  | 6 | 1059 | 0.028 | 126 | 93 | 49 | L_SS | 72 |
|  | 7 | 465 | 0.028 | 79 | 86 | 102 | R_CGC | 173 |
|  | 8 | 323 | 0.034 | 94 | 120 | 76 | Fx | 54 |
|  | 9 | 168 | 0.034 | 62 | 63 | 86 | R_PTR | 168 |
|  | 10 | 113 | 0.034 | 58 | 62 | 63 | R_ILF | 113 |
|  | 11 | 84 | 0.042 | 82 | 123 | 81 | R_ATR | 84 |
|  | 12 | 79 | 0.042 | 120 | 143 | 101 | L_ACR | 79 |
|  | 13 | 77 | 0.042 | 76 | 104 | 103 | BCC | 71 |
|  | 14 | 58 | 0.038 | 72 | 145 | 103 | R_ACR | 58 |
|  | 15 | 52 | 0.034 | 70 | 136 | 110 | R_ACR | 52 |
| twist | 1 | 31523 | 0.002 | 87 | 91 | 25 | R_PTR | 762 |
|  |  |  |  |  |  |  | L_PTR | 714 |
|  |  |  |  |  |  |  | L_RLIC | 634 |
|  |  |  |  |  |  |  | R_SLF | 634 |
|  |  |  |  |  |  |  | L_SLF | 592 |
|  | 2 | 98 | 0.034 | 61 | 139 | 80 | R_EC | 98 |
| HFS > HC | | | | | | | | |
| MD | 1 | 3708 | 0.028 | 65 | 74 | 31 | MCP | 638 |
|  |  |  |  |  |  |  | PCT | 134 |
|  |  |  |  |  |  |  | R_CST | 105 |
|  |  |  |  |  |  |  | R_ICP | 85 |
|  |  |  |  |  |  |  | L_CST | 66 |
| distortion | 1 | 2138 | 0.004 | 77 | 100 | 53 | R_PLIC | 535 |
|  |  |  |  |  |  |  | R_CP | 447 |
|  |  |  |  |  |  |  | R_ALIC | 341 |
|  |  |  |  |  |  |  | R_SCR | 156 |
|  |  |  |  |  |  |  | MCP | 126 |
|  | 2 | 1035 | 0.02 | 81 | 158 | 71 | GCC | 732 |
|  |  |  |  |  |  |  | R_ACR | 180 |
|  |  |  |  |  |  |  | L_ACR | 88 |
|  | 3 | 544 | 0.022 | 114 | 113 | 80 | L_PLIC | 377 |
|  |  |  |  |  |  |  | L_SCR | 64 |
|  | 4 | 275 | 0.028 | 65 | 108 | 100 | R_SCR | 189 |
|  | 5 | 111 | 0.036 | 106 | 104 | 61 | L_CP | 110 |
| splay | 1 | 23476 | 0.002 | 92 | 91 | 24 | GCC | 1071 |
|  |  |  |  |  |  |  | SCC | 981 |
|  |  |  |  |  |  |  | R_PTR | 808 |
|  |  |  |  |  |  |  | L_PTR | 801 |
|  |  |  |  |  |  |  | L_SLF | 713 |
| bend | 1 | 17830 | 0.002 | 98 | 86 | 38 | SCC | 2044 |
|  |  |  |  |  |  |  | R_PTR | 1000 |
|  |  |  |  |  |  |  | L_PTR | 992 |
|  |  |  |  |  |  |  | L_PLIC | 702 |
|  |  |  |  |  |  |  | R_RLIC | 617 |
|  | 2 | 2053 | 0.004 | 84 | 151 | 72 | GCC | 1121 |
|  |  |  |  |  |  |  | R_ACR | 415 |
|  |  |  |  |  |  |  | L_ACR | 364 |
|  | 3 | 265 | 0.048 | 55 | 77 | 46 |  |  |
|  | 4 | 128 | 0.048 | 103 | 76 | 51 |  |  |
|  | 5 | 66 | 0.048 | 77 | 93 | 33 | MCP | 66 |

Note: To briefly present the results, the top five white matter fiber regions with the highest number of voxels in each cluster were shown in the table. L: Left; R: Right.

Table S4: The results of TBSS between MS patients and HC in DTI metrics.

| Contrast group | Cluster index | Cluster size | *P_perm_* value | Peak MNI  coordinates (mm) | | | White matter location  (JHU-81 Atlas) | |
| --- | --- | --- | --- | --- | --- | --- | --- | --- |
|  |  |  |  | X | Y | Z | Regions | Voxel number |
| HC > MS | | | | | | | | |
| distortion | 1 | 371 | 0.034 | 107 | 88 | 108 | BCC | 105 |
|  |  |  |  |  |  |  | L_PCR | 103 |
|  |  |  |  |  |  |  | SCC | 67 |
|  | 2 | 188 | 0.042 | 61 | 108 | 106 | R_SCR | 88 |
|  | 3 | 142 | 0.044 | 120 | 101 | 106 | L_SLF | 35 |
| twist | 1 | 965 | 0.02 | 65 | 112 | 106 | BCC | 270 |
|  |  |  |  |  |  |  | SCC | 66 |
|  |  |  |  |  |  |  | R_SCR | 243 |
|  | 2 | 418 | 0.02 | 110 | 87 | 102 | BCC | 113 |
|  |  |  |  |  |  |  | L_PCR | 109 |
|  |  |  |  |  |  |  | SCC | 91 |
|  | 3 | 93 | 0.038 | 114 | 68 | 96 |  |  |
|  | 4 | 77 | 0.042 | 86 | 116 | 98 | BCC | 77 |

Note: L: Left; R: Right.

Table S5: The results of TBSS of One-way ANOVA among three facial diseases in DTI metrics.

| Contrast group | Cluster index | Cluster size | *P_perm_* value | Peak MNI  coordinates (mm) | | | White matter location  (JHU-81 Atlas) | |
| --- | --- | --- | --- | --- | --- | --- | --- | --- |
|  |  |  |  | X | Y | Z | Regions | Voxel number |
| FA | 1 | 1070 | 0.024 | 96 | 89 | 89 | SCC | 700 |
|  |  |  |  |  |  |  | BCC | 370 |
| MD | 1 | 6985 | 0.002 | 115 | 106 | 100 | L_SLF | 928 |
|  |  |  |  |  |  |  | L_SCR | 495 |
|  |  |  |  |  |  |  | L_PLIC | 435 |
|  |  |  |  |  |  |  | L_RLIC | 404 |
|  |  |  |  |  |  |  | L_PCR | 363 |
| distortion | 1 | 11336 | 0.006 | 121 | 96 | 75 | L_SCR | 523 |
|  |  |  |  |  |  |  | L_RLIC | 472 |
|  |  |  |  |  |  |  | L_PTR | 465 |
|  |  |  |  |  |  |  | R_PTR | 415 |
|  |  |  |  |  |  |  | R_RLIC | 319 |
| splay | 1 | 6936 | 0.004 | 60 | 115 | 60 | R_PLIC | 334 |
|  |  |  |  |  |  |  | R_RLIC | 316 |
|  |  |  |  |  |  |  | R_SCR | 307 |
|  |  |  |  |  |  |  | L_RLIC | 296 |
|  |  |  |  |  |  |  | L_PLIC | 237 |
| bend | 1 | 3767 | 0.012 | 71 | 107 | 67 | R_SCR | 414 |
|  |  |  |  |  |  |  | R_PLIC | 285 |
|  |  |  |  |  |  |  | L_RLIC | 253 |
|  |  |  |  |  |  |  | L_PLIC | 223 |
|  |  |  |  |  |  |  | L_CP | 214 |
|  | 2 | 3436 | 0.04 | 73 | 83 | 80 | BCC | 822 |
|  |  |  |  |  |  |  | L_SCR | 619 |
|  |  |  |  |  |  |  | SCC | 495 |
|  |  |  |  |  |  |  | R_SCR | 327 |
|  |  |  |  |  |  |  | L_PCR | 258 |
| twist | 1 | 3713 | 0.008 | 53 | 120 | 48 | R_PTR | 433 |
|  |  |  |  |  |  |  | R_RLIC | 340 |
|  |  |  |  |  |  |  | R_CP | 264 |
|  |  |  |  |  |  |  | SCC | 221 |
|  |  |  |  |  |  |  | R_PCR | 173 |
|  | 2 | 2612 | 0.008 | 118 | 101 | 63 | L_PLIC | 515 |
|  |  |  |  |  |  |  | L_PTR | 434 |
|  |  |  |  |  |  |  | L_Fx/ST | 211 |
|  |  |  |  |  |  |  | SCC | 138 |
|  |  |  |  |  |  |  | L_EC | 130 |
|  | 3 | 1719 | 0.01 | 102 | 84 | 34 | L_CP | 351 |
|  |  |  |  |  |  |  | MCP | 174 |
|  |  |  |  |  |  |  | L_ICP | 124 |
|  |  |  |  |  |  |  | L_SCP | 120 |
|  | 4 | 296 | 0.03 | 68 | 75 | 37 | MCP | 128 |
|  |  |  |  |  |  |  | R_ICP | 99 |

Note: To briefly present the results, the top five white matter fiber regions with the highest number of voxels in each cluster were shown in the table. L: Left; R: Right.

Table S6: The results of TBSS between FP and MS patients in DTI metrics.

| Contrast group | Cluster index | Cluster size | *P_perm_* value | Peak MNI  coordinates (mm) | | | White matter location  (JHU-81 Atlas) | |
| --- | --- | --- | --- | --- | --- | --- | --- | --- |
|  |  |  |  | X | Y | Z | Regions | Voxel number |
| MS > FP | | | | | | | | |
| FA | 1 | 17506 | 0.018 | 62 | 79 | 32 | SCC | 1632 |
|  |  |  |  |  |  |  | MCP | 778 |
|  |  |  |  |  |  |  | R_SLF | 675 |
|  |  |  |  |  |  |  | L_ALIC | 632 |
|  |  |  |  |  |  |  | R_RLIC | 569 |
|  | 2 | 1148 | 0.036 | 77 | 136 | 72 | R_ALIC | 458 |
|  |  |  |  |  |  |  | R_ACR | 308 |
|  |  |  |  |  |  |  | R_PLIC | 293 |
|  | 3 | 587 | 0.046 | 86 | 145 | 89 | BCC | 559 |
| MD | 1 | 2232 | 0.016 | 72 | 166 | 66 | R_ACR | 699 |
|  |  |  |  |  |  |  | GCC | 397 |
|  | 2 | 1756 | 0.016 | 105 | 161 | 62 | L_ACR | 735 |
|  |  |  |  |  |  |  | GCC | 181 |
| distortion | 1 | 431 | 0.022 | 122 | 104 | 65 | L_RLIC | 208 |
| bend | 1 | 127 | 0.038 | 122 | 110 | 120 | L_SLF | 103 |
|  | 2 | 76 | 0.042 | 122 | 153 | 90 |  |  |
| twist | 1 | 27902 | 0.002 | 132 | 117 | 43 | BCC | 1315 |
|  |  |  |  |  |  |  | R_PTR | 728 |
|  |  |  |  |  |  |  | SCC | 677 |
|  |  |  |  |  |  |  | L_RLIC | 652 |
|  |  |  |  |  |  |  | R_SLF | 468 |
| FP > MS | | | | | | | | |
| MD | 1 | 11909 | 0.006 | 100 | 122 | 63 | MCP | 1412 |
|  |  |  |  |  |  |  | L_PLIC | 572 |
|  |  |  |  |  |  |  | R_CP | 441 |
|  |  |  |  |  |  |  | L_CP | 399 |
|  |  |  |  |  |  |  | R_PLIC | 390 |
| distortion | 1 | 6813 | 0.004 | 99 | 107 | 49 | L_SCR | 712 |
|  |  |  |  |  |  |  | R_SCR | 675 |
|  |  |  |  |  |  |  | L_PLIC | 569 |
|  |  |  |  |  |  |  | R_PLIC | 507 |
|  |  |  |  |  |  |  | R_CP | 417 |
|  | 2 | 503 | 0.018 | 97 | 156 | 72 | GCC | 350 |
|  |  |  |  |  |  |  | L_ACR | 121 |
|  | 3 | 203 | 0.04 | 75 | 160 | 79 | GCC | 127 |
|  |  |  |  |  |  |  | R_ACR | 73 |
|  | 4 | 188 | 0.03 | 117 | 142 | 98 | L_SCR | 59 |
| splay | 1 | 28730 | 0.002 | 85 | 93 | 29 | SCC | 1470 |
|  |  |  |  |  |  |  | GCC | 1201 |
|  |  |  |  |  |  |  | BCC | 1166 |
|  |  |  |  |  |  |  | R_SLF | 947 |
|  |  |  |  |  |  |  | L_PTR | 863 |
| bend | 1 | 26200 | 0.002 | 68 | 80 | 33 | SCC | 1866 |
|  |  |  |  |  |  |  | L_PTR | 1056 |
|  |  |  |  |  |  |  | R_PTR | 958 |
|  |  |  |  |  |  |  | L_PLIC | 728 |
|  |  |  |  |  |  |  | R_SLF | 701 |
|  | 2 | 409 | 0.034 | 96 | 157 | 79 | GCC | 336 |
|  |  |  |  |  |  |  | L_ACR | 59 |
|  | 3 | 336 | 0.042 | 86 | 150 | 71 | GCC | 276 |
|  | 4 | 83 | 0.042 | 107 | 84 | 37 | MCP | 83 |

Note: To briefly present the results, the top five white matter fiber regions with the highest number of voxels in each cluster were shown in the table. L: Left; R: Right.

Table S7: The results of TBSS between HFS and MS patients DTI metrics.

| Contrast group | Cluster index | Cluster size | *P_perm_* value | Peak MNI  coordinates (mm) | | | White matter location  (JHU-81 Atlas) | |
| --- | --- | --- | --- | --- | --- | --- | --- | --- |
|  |  |  |  | X | Y | Z | Regions | Voxel number |
| MS > HFS | | | | | | | | |
| FA | 1 | 34869 | 0.002 | 76 | 88 | 33 | SCC | 2088 |
|  |  |  |  |  |  |  | GCC | 1643 |
|  |  |  |  |  |  |  | MCP | 1538 |
|  |  |  |  |  |  |  | BCC | 1395 |
|  |  |  |  |  |  |  | R_PTR | 898 |
|  | 2 | 510 | 0.044 | 54 | 161 | 79 | R_SLF | 510 |
|  | 3 | 351 | 0.048 | 70 | 106 | 121 | R_SCR | 351 |
|  | 4 | 325 | 0.046 | 42 | 126 | 96 | R_SLF | 325 |
| distortion | 1 | 18507 | 0.006 | 121 | 100 | 66 | BCC | 1419 |
|  |  |  |  |  |  |  | L_ACR | 528 |
|  |  |  |  |  |  |  | L_RLIC | 459 |
|  |  |  |  |  |  |  | R_ACR | 365 |
|  |  |  |  |  |  |  | L_PTR | 348 |
|  | 2 | 139 | 0.028 | 61 | 98 | 68 | R_Fx/ST | 66 |
| bend | 1 | 1342 | 0.03 | 55 | 132 | 92 | R_ACR | 173 |
|  |  |  |  |  |  |  | R_SLF | 54 |
|  | 2 | 470 | 0.026 | 112 | 163 | 86 | BCC | 99 |
|  |  |  |  |  |  |  | L_ACR | 229 |
|  | 3 | 368 | 0.034 | 107 | 126 | 109 | BCC | 191 |
|  |  |  |  |  |  |  | L_SCR | 147 |
|  | 4 | 334 | 0.038 | 72 | 105 | 107 | BCC | 157 |
|  |  |  |  |  |  |  | R_SCR | 155 |
|  | 5 | 146 | 0.044 | 111 | 166 | 92 | L_ATR | 146 |
|  | 6 | 87 | 0.048 | 121 | 152 | 88 | L_ATR | 87 |
| twist | 1 | 30806 | 0.002 | 132 | 117 | 43 | BCC | 1569 |
|  |  |  |  |  |  |  | R_PTR | 696 |
|  |  |  |  |  |  |  | L_RLIC | 648 |
|  |  |  |  |  |  |  | L_PTR | 630 |
|  |  |  |  |  |  |  | R_SLF | 592 |
| HFS > MS | | | | | | | | |
| FA | 1 | 863 | 0.038 | 98 | 79 | 49 | L_SCP |  |
| MD | 1 | 13421 | 0.006 | 77 | 85 | 32 | MCP | 1658 |
|  |  |  |  |  |  |  | L_ALIC | 409 |
|  |  |  |  |  |  |  | L_PLIC | 383 |
|  |  |  |  |  |  |  | L_SCR | 382 |
|  |  |  |  |  |  |  | R_CP | 266 |
| distortion | 1 | 4335 | 0.002 | 78 | 97 | 48 | R_PLIC | 564 |
|  |  |  |  |  |  |  | R_CP | 495 |
|  |  |  |  |  |  |  | R_SCR | 464 |
|  |  |  |  |  |  |  | R_ALIC | 420 |
|  |  |  |  |  |  |  | MCP | 270 |
|  | 2 | 2398 | 0.002 | 100 | 109 | 51 | L_PLIC | 592 |
|  |  |  |  |  |  |  | L_SCR | 481 |
|  |  |  |  |  |  |  | L_CP | 449 |
|  |  |  |  |  |  |  | L_ALIC | 243 |
|  |  |  |  |  |  |  | L_EC | 189 |
|  | 3 | 1538 | 0.004 | 103 | 159 | 71 | GCC | 890 |
|  |  |  |  |  |  |  | L_ACR | 340 |
|  |  |  |  |  |  |  | R_ACR | 226 |
|  | 4 | 91 | 0.044 | 113 | 155 | 70 | L_ACR | 91 |
|  | 5 | 64 | 0.046 | 120 | 135 | 76 | L_EC | 62 |
| splay | 1 | 29100 | 0.002 | 86 | 88 | 29 | GCC | 1226 |
|  |  |  |  |  |  |  | MCP | 1037 |
|  |  |  |  |  |  |  | SCC | 984 |
|  |  |  |  |  |  |  | R_SLF | 867 |
|  |  |  |  |  |  |  | R_PTR | 860 |
|  | 2 | 775 | 0.026 | 57 | 81 | 41 |  |  |
|  | 3 | 209 | 0.032 | 108 | 146 | 110 |  |  |
| bend | 1 | 24316 | 0.008 | 67 | 76 | 30 | SCC | 1874 |
|  |  |  |  |  |  |  | GCC | 1201 |
|  |  |  |  |  |  |  | L_PTR | 983 |
|  |  |  |  |  |  |  | R_PTR | 968 |
|  |  |  |  |  |  |  | L_PLIC | 717 |
|  | 2 | 1084 | 0.01 | 63 | 108 | 105 | R_SLF | 516 |
|  |  |  |  |  |  |  | R_SCR | 155 |
|  | 3 | 624 | 0.026 | 102 | 62 | 52 |  |  |
|  | 4 | 116 | 0.048 | 67 | 85 | 111 |  |  |
| twist | 1 | 393 | 0.032 | 121 | 124 | 84 | L_SCR | 170 |
|  |  |  |  |  |  |  | L_EC | 74 |

Note: To briefly present the results, the top five white matter fiber regions with the highest number of voxels in each cluster were shown in the table. L: Left; R: Right.

Table S8: The Results of TBSS between HC_1 and HC_2 patients in DTI metrics.

| Contrast group | Cluster index | Cluster size | *P_perm_* value | Peak MNI  coordinates (mm) | | | White matter location  (JHU-81 Atlas) | |
| --- | --- | --- | --- | --- | --- | --- | --- | --- |
|  |  |  |  | X | Y | Z | Regions | Voxel number |
| HC_1 > HC_2 | | | | | | | | |
| FA | 1 | 1662 | 0.03 | 19 | 11 | 41 | R_ACR | 26 |
|  |  |  |  |  |  |  | R_SCR | 26 |
|  | 2 | 1022 | 0.036 | -18 | 18 | 37 | L_SCR | 72 |
|  | 3 | 63 | 0.046 | 9 | 26 | -5 | GCC | 63 |
|  | 4 | 59 | 0.046 | -8 | 24 | -5 | GCC | 51 |
| HC_2 > HC_1 | | | | | | | | |
| twist | 1 | 5040 | 0.008 | 13 | -27 | 27 | BCC | 1765 |
|  |  |  |  |  |  |  | GCC | 656 |
|  |  |  |  |  |  |  | SCC | 439 |
|  |  |  |  |  |  |  | L_ACR | 283 |
|  |  |  |  |  |  |  | R_SCR | 213 |
| distortion | 1 | 3292 | 0.02 | 8 | 17 | 20 | BCC | 1428 |
|  |  |  |  |  |  |  | GCC | 512 |
|  |  |  |  |  |  |  | L_ACR | 215 |
|  |  |  |  |  |  |  | R_ACR | 174 |
|  |  |  |  |  |  |  | SCC | 141 |

Note: To briefly present the results, the top five white matter fiber regions with the highest number of voxels in each cluster were shown in the table. L: Left; R: Right.

Table S9: Results of classification using AutoGluon

| Feature | | Accuracy | Sensitivity | Specificity | ROC_AUC |
| --- | --- | --- | --- | --- | --- |
| FP v.s. HC | |  |  |  |  |
|  | FA+MD+DFA | 0.5783 | 0.2121 | 0.82 | 0.6183 |
|  | DFA | 0.6265 | 0.1818 | 0.92 | 0.6333 |
|  | FA+MD | 0.6506 | 0.2727 | 0.9 | 0.7317 |
| HFS v.s. HC | |  |  |  |  |
|  | FA+MD+DFA | 0.6556 | 0.4848 | 0.7544 | 0.7328 |
|  | DFA | 0.7111 | 0.5455 | 0.807 | 0.755 |
|  | FA+MD | 0.6556 | 0.3333 | 0.8421 | 0.6944 |
| MS v.s. HC | |  |  |  |  |
|  | FA+MD+DFA | 0.5738 | 0.4667 | 0.6774 | 0.5639 |
|  | DFA | 0.5246 | 0.4333 | 0.6129 | 0.5056 |
|  | FA+MD | 0.4918 | 0.3667 | 0.6129 | 0.5417 |
| FP v.s. MS | |  |  |  |  |
|  | FA+MD+DFA | 0.7778 | 0.92 | 0.5484 | 0.9683 |
|  | DFA | 0.7037 | 0.86 | 0.4516 | 0.86 |
|  | FA+MD | 0.7407 | 0.92 | 0.4516 | 0.9 |
| HFS v.s. MS | |  |  |  |  |
|  | FA+MD+DFA | 0.6364 | 0.129 | 0.9123 | 0.8078 |
|  | DFA | 0.6932 | 0.3226 | 0.8947 | 0.7761 |
|  | FA+MD | 0.6591 | 0.2258 | 0.8947 | 0.7578 |
| FP v.s. HFS | |  |  |  |  |
|  | FA+MD+DFA | 0.5981 | 0.52 | 0.6667 | 0.5667 |
|  | DFA | 0.5047 | 0.32 | 0.6667 | 0.526 |
|  | FA+MD | 0.6449 | 0.52 | 0.7544 | 0.6773 |

Table S10: Results of classification using SVM, Logistic Regression and Random Forests

| Classifier | Task | Feature | Accuracy | Sensitivity | Specificity | ROC_AUC |
| --- | --- | --- | --- | --- | --- | --- |
| SVM | FP v.s. HC | FA+MD+DFA | 0.5783 | 0.6061 | 0.56 | 0.5745 |
|  |  | DFA | 0.5301 | 0.5455 | 0.52 | 0.4939 |
|  |  | FA+MD | 0.6747 | 0.3333 | 0.9 | 0.7161 |
|  | HFS v.s. HC | FA+MD+DFA | 0.6444 | 0.4545 | 0.7544 | 0.7517 |
|  |  | DFA | 0.6222 | 0.5152 | 0.6842 | 0.7129 |
|  |  | FA+MD | 0.6556 | 0.2121 | 0.9123 | 0.6821 |
|  | MS v.s. HC | FA+MD+DFA | 0.5574 | 0.4333 | 0.6774 | 0.3925 |
|  |  | DFA | 0.541 | 0.4 | 0.6774 | 0.3559 |
|  |  | FA+MD | 0.5738 | 0.5 | 0.6452 | 0.3548 |
|  | FP v.s. HFS | FA+MD+DFA | 0.5514 | 0.36 | 0.7193 | 0.4653 |
|  |  | DFA | 0.5047 | 0.34 | 0.6491 | 0.4323 |
|  |  | FA+MD | 0.6355 | 0.4 | 0.8421 | 0.6246 |
|  | FP v.s. MS | FA+MD+DFA | 0.8519 | 0.9 | 0.7742 | 0.9277 |
|  |  | DFA | 0.7654 | 0.8 | 0.7097 | 0.8626 |
|  |  | FA+MD | 0.7778 | 0.96 | 0.4839 | 0.8968 |
|  | HFS v.s. MS | FA+MD+DFA | 0.7045 | 0.4516 | 0.8421 | 0.7753 |
|  |  | DFA | 0.6932 | 0.4516 | 0.8246 | 0.7804 |
|  |  | FA+MD | 0.6705 | 0.2258 | 0.9123 | 0.7861 |
| Logistic Regression | FP v.s. HC | FA+MD+DFA | 0.5663 | 0.5152 | 0.6 | 0.663 |
|  |  | DFA | 0.5542 | 0.4545 | 0.62 | 0.6152 |
|  |  | FA+MD | 0.6145 | 0.5152 | 0.68 | 0.6903 |
|  | HFS v.s. HC | FA+MD+DFA | 0.6889 | 0.5758 | 0.7544 | 0.7544 |
|  |  | DFA | 0.6444 | 0.4848 | 0.7368 | 0.7144 |
|  |  | FA+MD | 0.6778 | 0.5758 | 0.7368 | 0.748 |
|  | MS v.s. HC | FA+MD+DFA | 0.4754 | 0.4333 | 0.5161 | 0.4538 |
|  |  | DFA | 0.4918 | 0.4667 | 0.5161 | 0.4806 |
|  |  | FA+MD | 0.5082 | 0.4667 | 0.5484 | 0.5043 |
|  | FP v.s. HFS | FA+MD+DFA | 0.5981 | 0.58 | 0.614 | 0.6565 |
|  |  | DFA | 0.486 | 0.46 | 0.5088 | 0.513 |
|  |  | FA+MD | 0.6262 | 0.62 | 0.6316 | 0.713 |
|  | FP v.s. MS | FA+MD+DFA | 0.8765 | 0.9 | 0.8387 | 0.9432 |
|  |  | DFA | 0.8148 | 0.88 | 0.7097 | 0.8884 |
|  |  | FA+MD | 0.8272 | 0.88 | 0.7419 | 0.9155 |
|  | HFS v.s. MS | FA+MD+DFA | 0.7273 | 0.5806 | 0.807 | 0.8308 |
|  |  | DFA | 0.7273 | 0.6129 | 0.7895 | 0.7838 |
|  |  | FA+MD | 0.7614 | 0.6129 | 0.8421 | 0.8002 |
| Random Forests | FP v.s. HC | FA+MD+DFA | 0.5663 | 0.3939 | 0.68 | 0.6894 |
|  |  | DFA | 0.5904 | 0.5758 | 0.6 | 0.6824 |
|  |  | FA+MD | 0.6386 | 0.4545 | 0.76 | 0.6745 |
|  | HFS v.s. HC | FA+MD+DFA | 0.6778 | 0.4242 | 0.8246 | 0.7578 |
|  |  | DFA | 0.6889 | 0.4545 | 0.8246 | 0.7924 |
|  |  | FA+MD | 0.7111 | 0.4848 | 0.8421 | 0.7767 |
|  | MS v.s. HC | FA+MD+DFA | 0.5082 | 0.3 | 0.7097 | 0.522 |
|  |  | DFA | 0.4918 | 0.3333 | 0.6452 | 0.4978 |
|  |  | FA+MD | 0.541 | 0.4333 | 0.6452 | 0.5446 |
|  | FP v.s. HFS | FA+MD+DFA | 0.5514 | 0.42 | 0.6667 | 0.5786 |
|  |  | DFA | 0.5701 | 0.46 | 0.6667 | 0.593 |
|  |  | FA+MD | 0.6075 | 0.5 | 0.7018 | 0.6472 |
|  | FP v.s. MS | FA+MD+DFA | 0.7901 | 0.9 | 0.6129 | 0.8797 |
|  |  | DFA | 0.7407 | 0.82 | 0.6129 | 0.8287 |
|  |  | FA+MD | 0.8519 | 0.88 | 0.8065 | 0.9126 |
|  | HFS v.s. MS | FA+MD+DFA | 0.7273 | 0.5806 | 0.807 | 0.8028 |
|  |  | DFA | 0.7273 | 0.6452 | 0.7719 | 0.8031 |
|  |  | FA+MD | 0.6818 | 0.3548 | 0.8596 | 0.7569 |
